# Supplementary material for: Species-specific sensitivity to TGFβ signaling and changes to the Mmp13 promoter underlie avian jaw development and evolution
Source: eLife. 2022 Jun 6;11:e66005. doi: 10.7554/eLife.66005 (PMC9246370; doi:10.7554/eLife.66005)

Chameleon Duo Ladder C31 Q31 D31 C34 Q34 D34

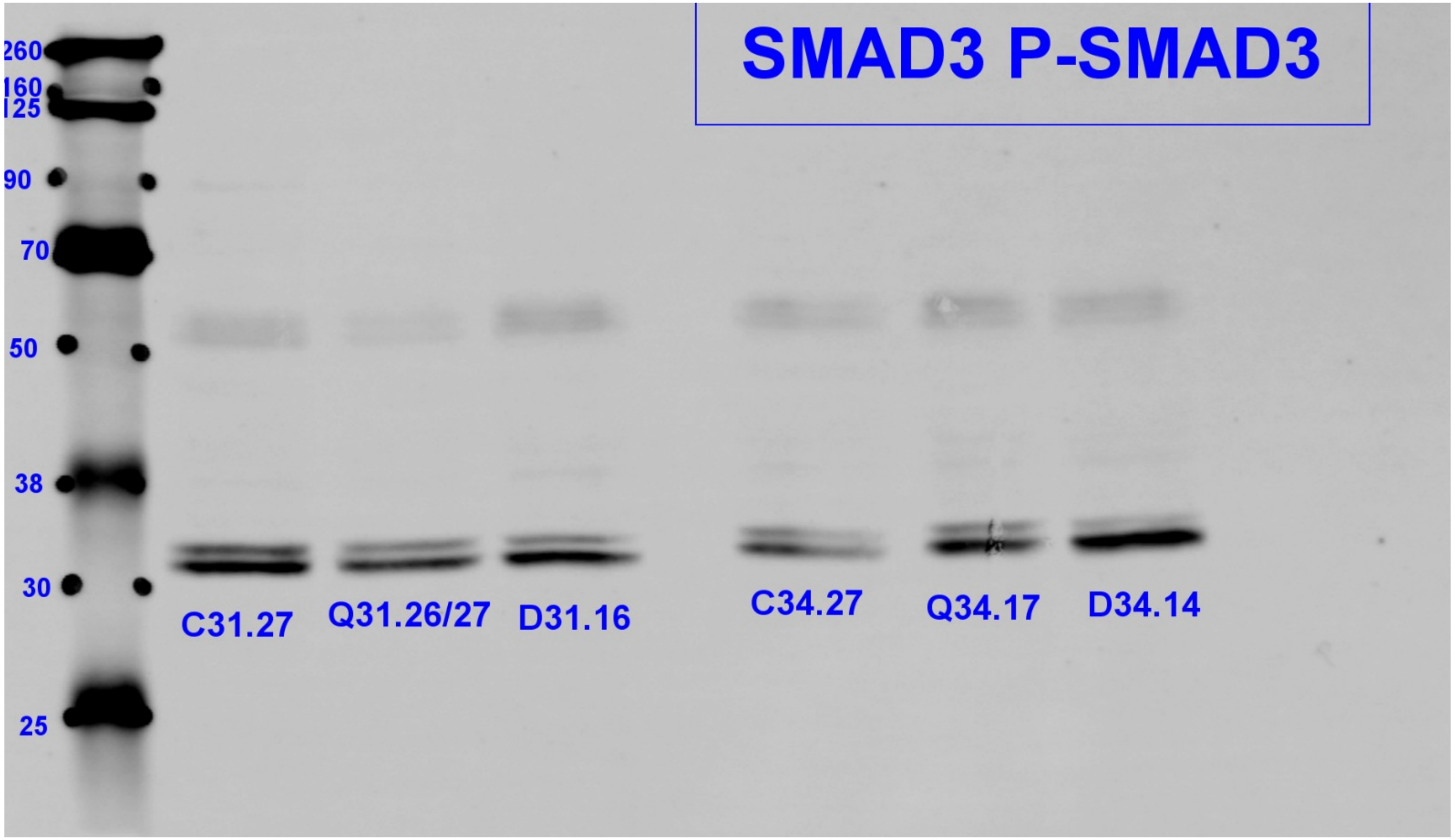

Figure 2-figure supplement 2A-source data 1 (β-Actin)

Chameleon Duo Ladder C31 Q31 D31 C34 Q34 D34

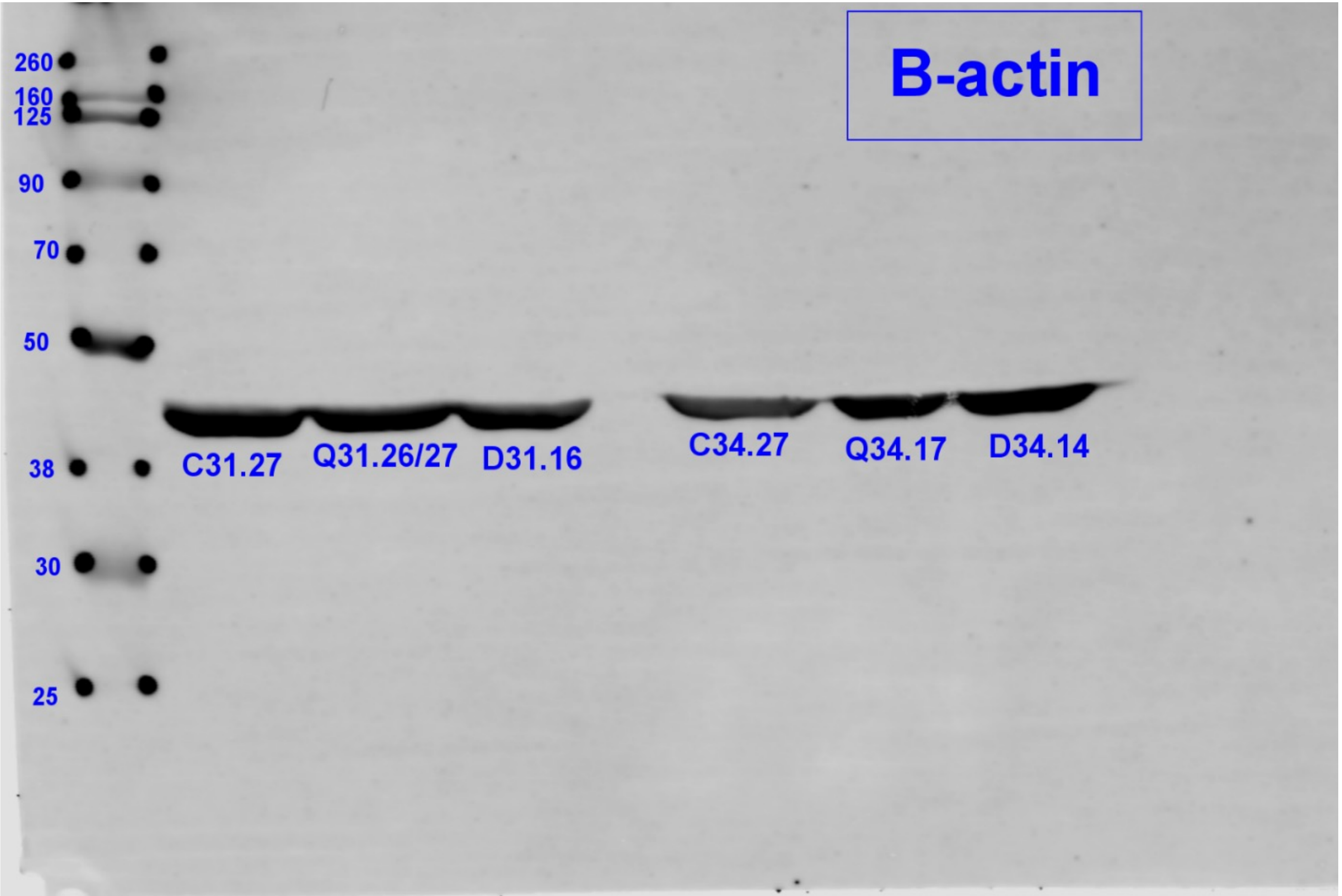

Figure 2-figure supplement 2A-source data 1 (pSMAD3)

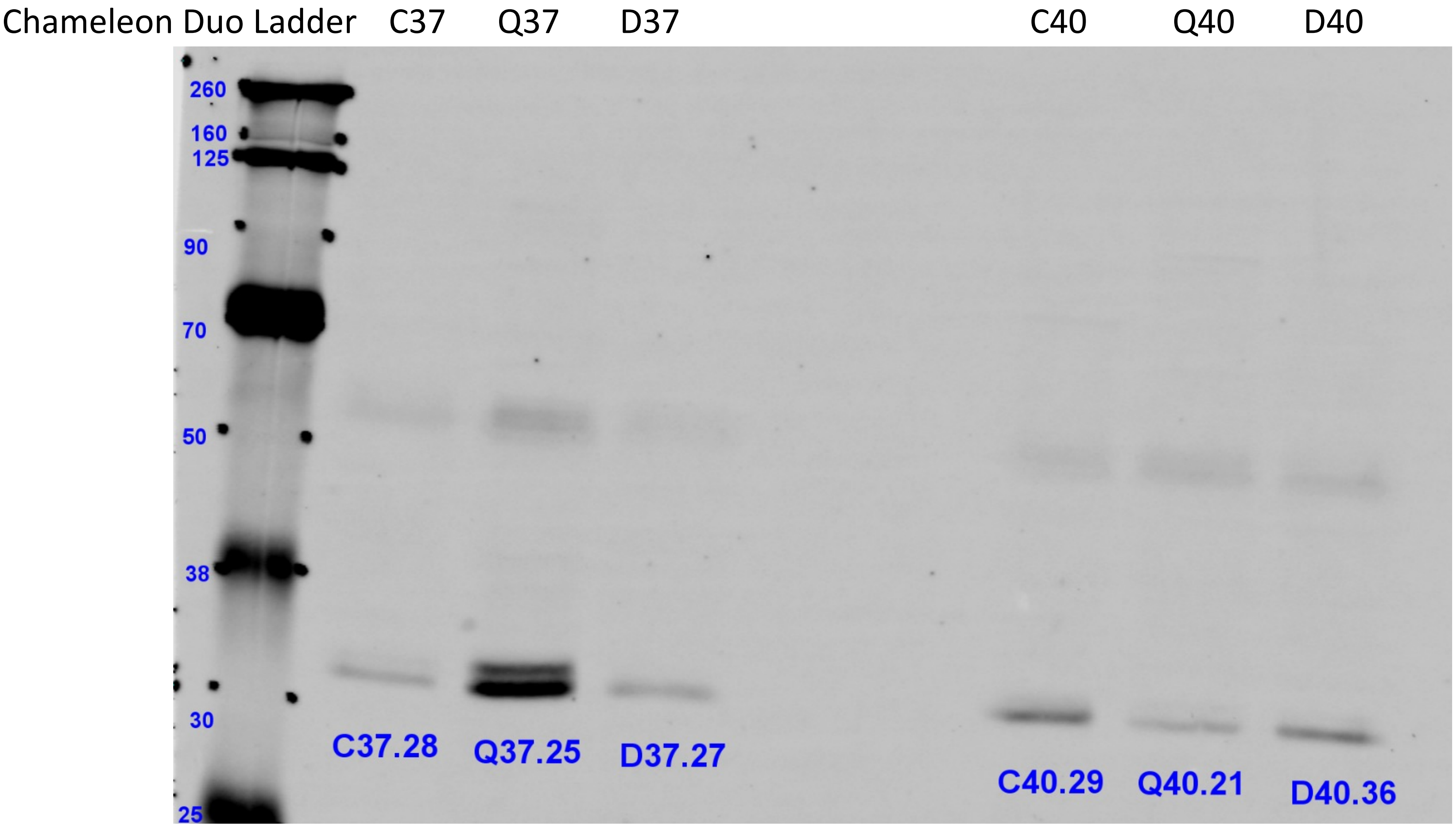

Figure 2-figure supplement 2A-source data 1 (β-Actin)

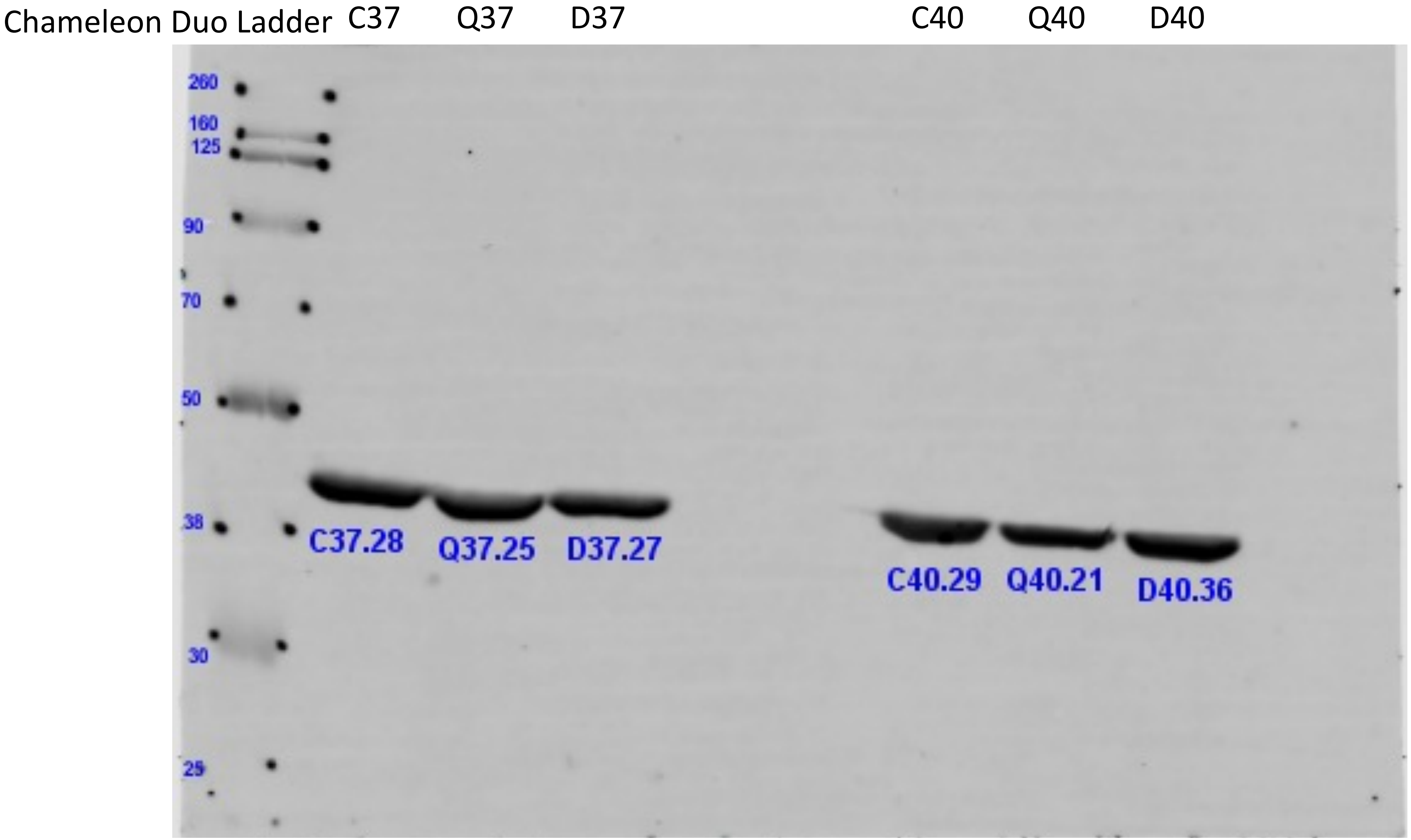

Supplement: Figure 2—figure supplement 2—source data 1. [file elife-66005-fig2-figsupp2-data1.pdf]
