# Supplementary material for: Species-specific sensitivity to TGFβ signaling and changes to the Mmp13 promoter underlie avian jaw development and evolution
Source: eLife. 2022 Jun 6;11:e66005. doi: 10.7554/eLife.66005 (PMC9246370; doi:10.7554/eLife.66005)

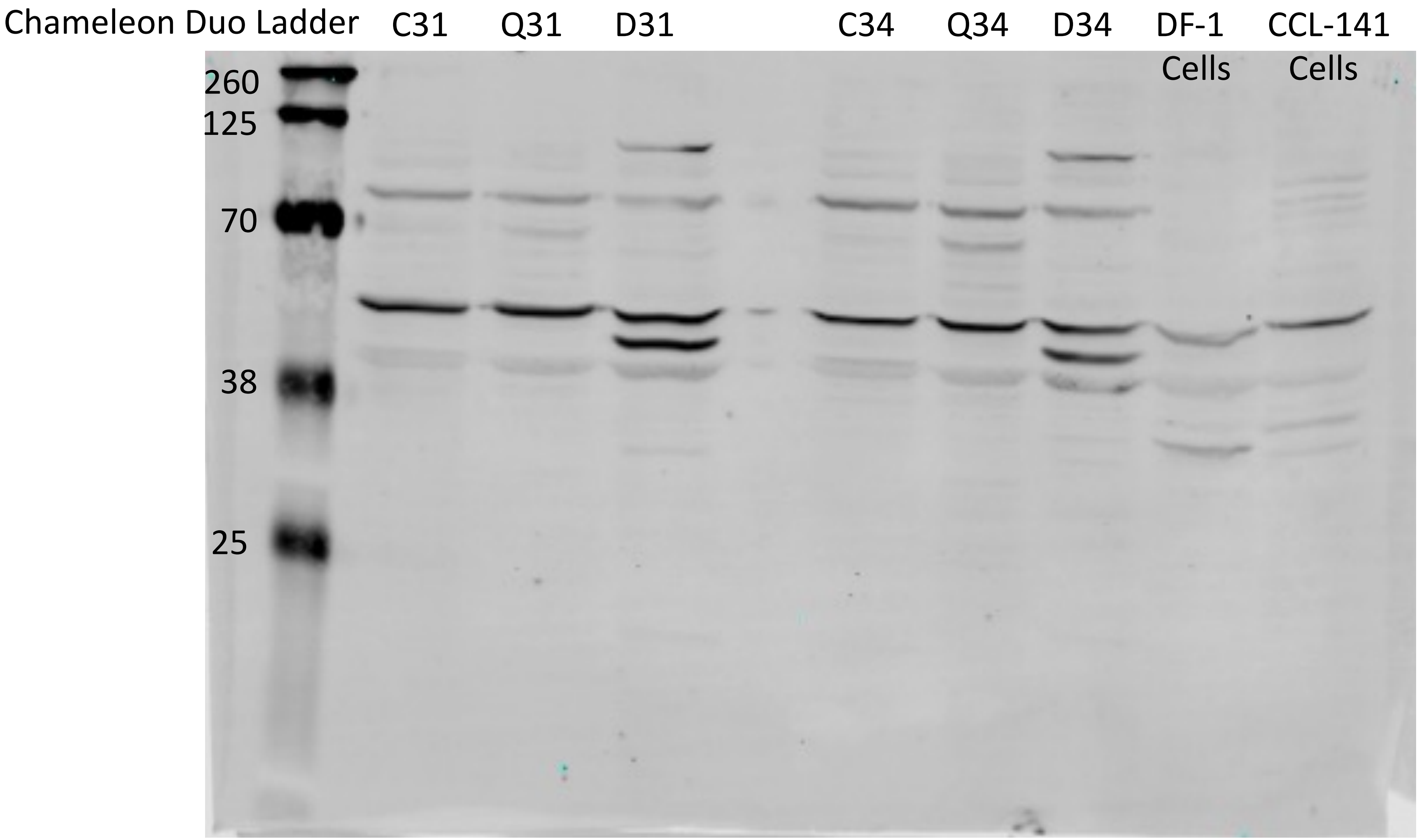

Chameleon Duo Ladder
C31
Q31
D31
C34
Q34
D34
DF-1 Cells
CCL-141 Cells
260
125
70
38
25

Figure 2-figure supplement 2B-source data 1 (β-Actin)

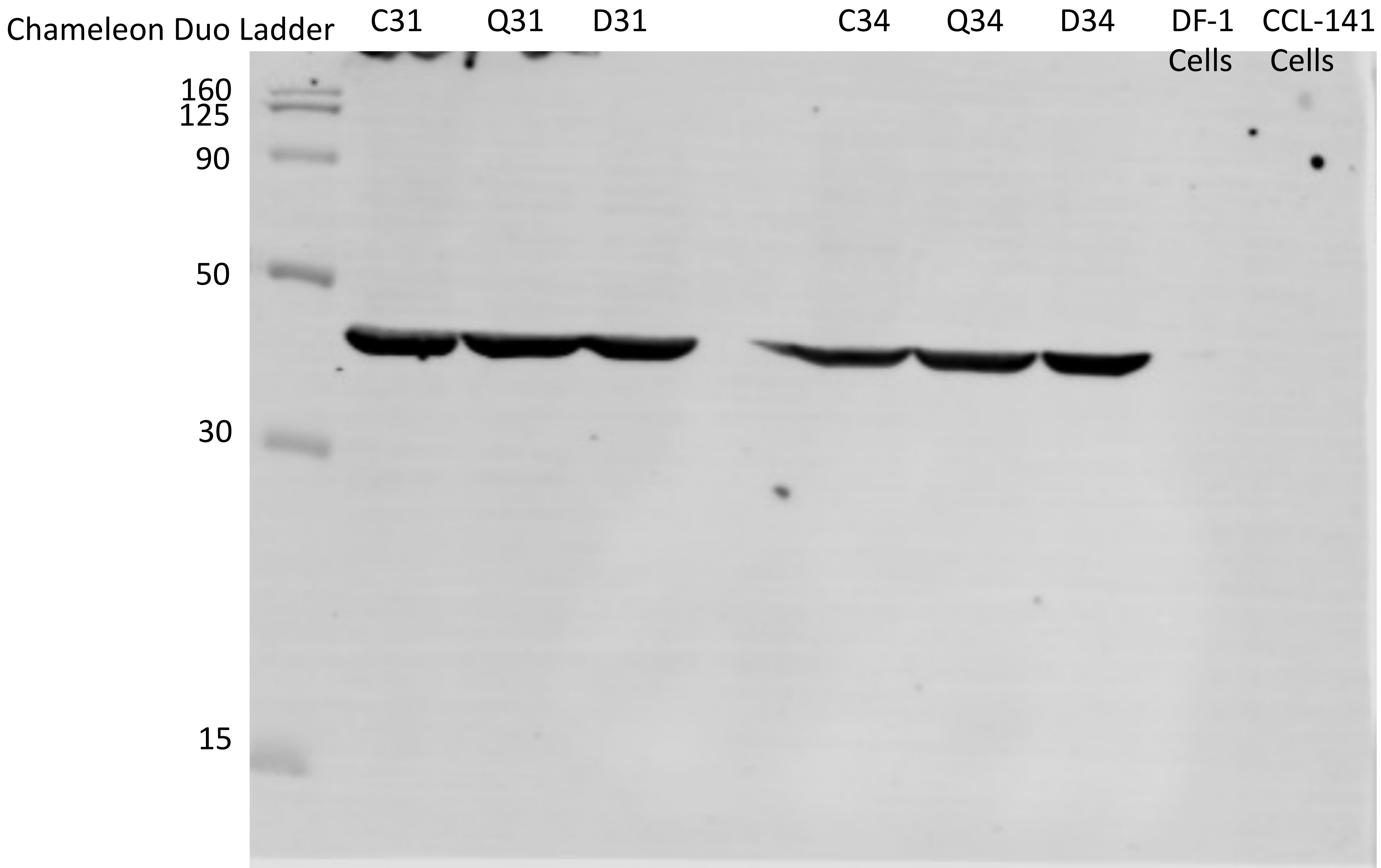

Figure 2-figure supplement 2B-source data 1 (MMP13)

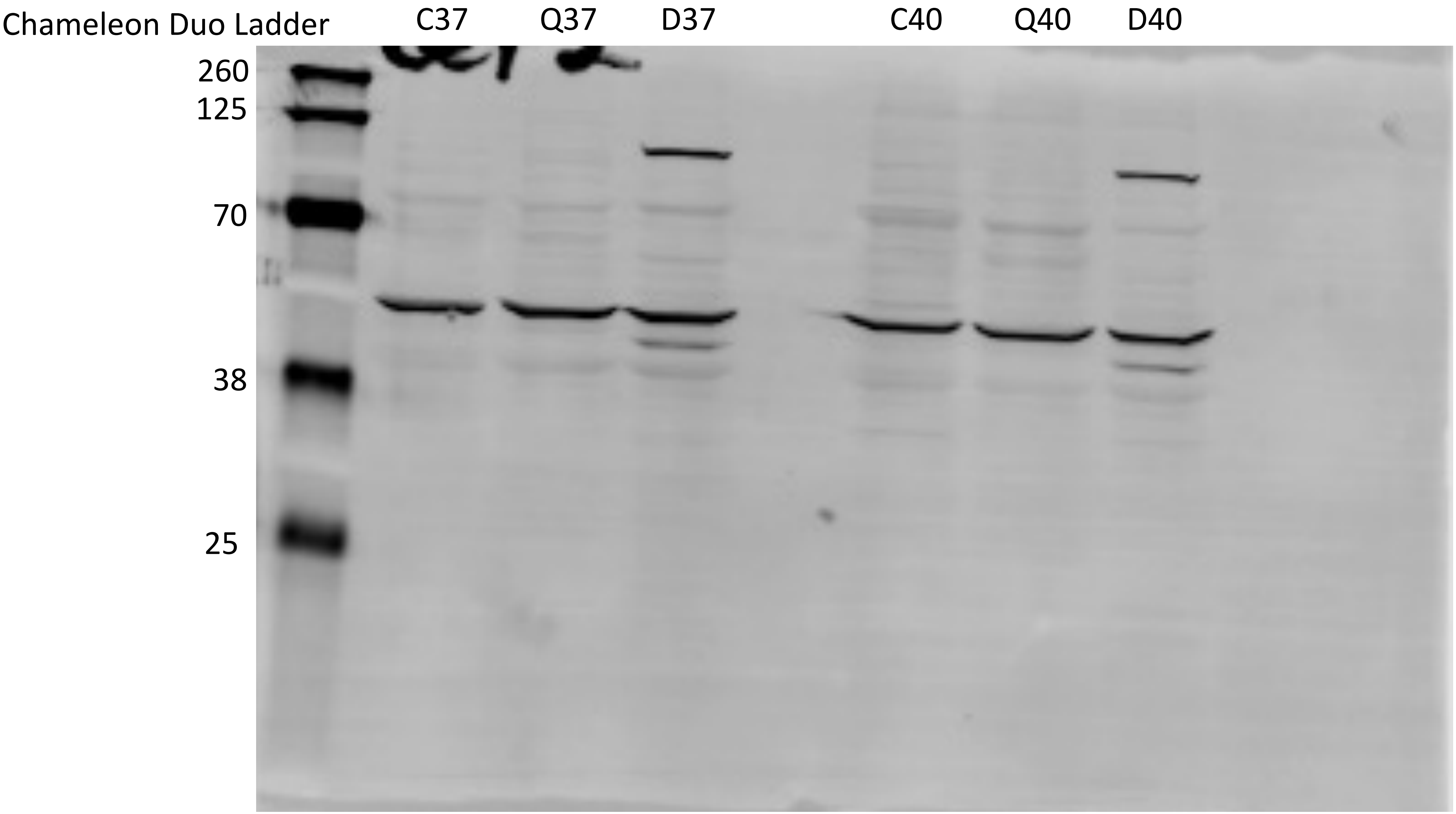

Figure 2-figure supplement 2B-source data 1 (β-Actin)

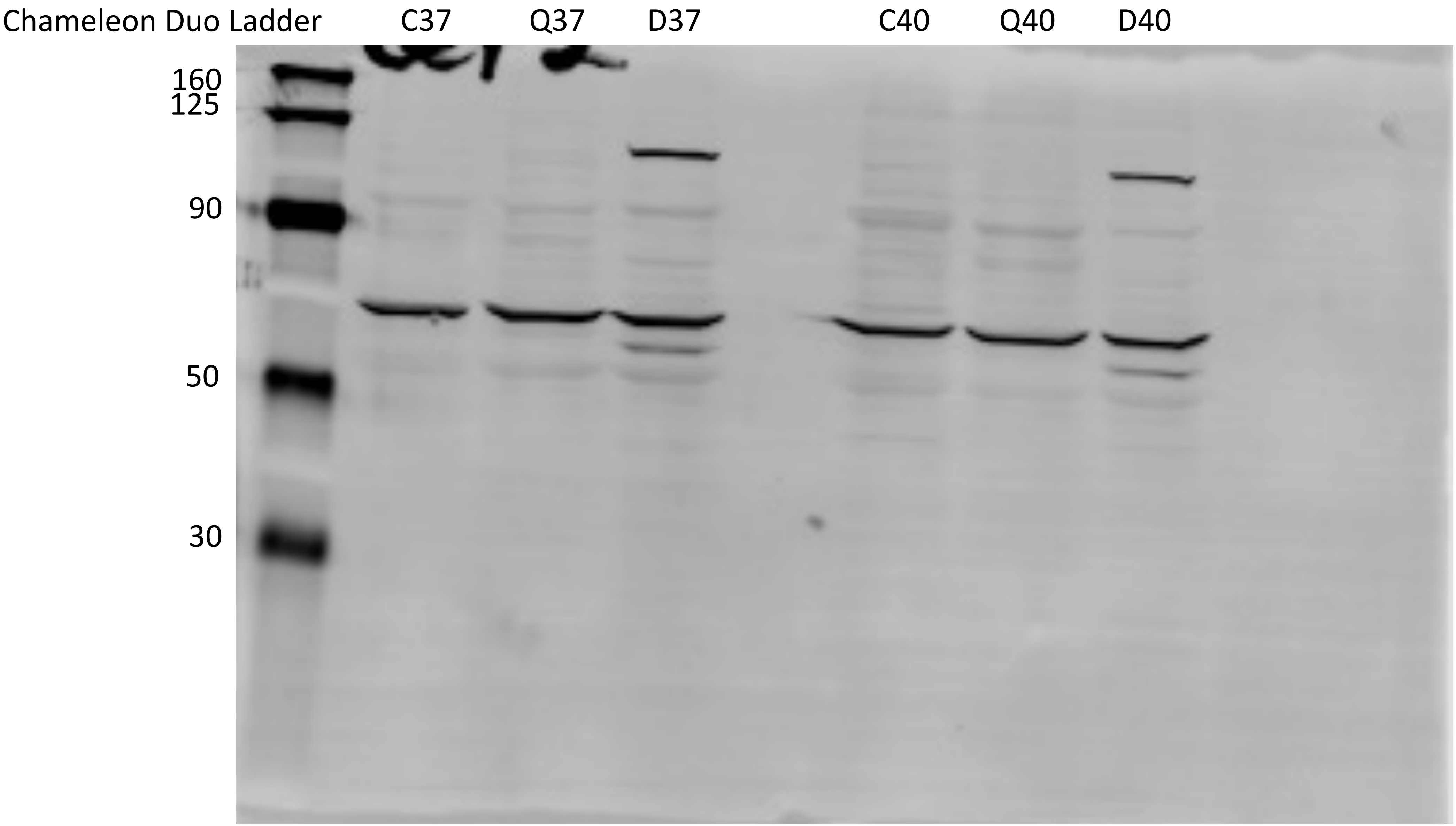

Supplement: Figure 2—figure supplement 2—source data 2. [file elife-66005-fig2-figsupp2-data2.pdf]
