# Supplementary figures and images for: Species-specific sensitivity to TGFβ signaling and changes to the Mmp13 promoter underlie avian jaw development and evolution
Source: eLife. 2022 Jun 6;11:e66005. doi: 10.7554/eLife.66005 (PMC9246370; doi:10.7554/eLife.66005)

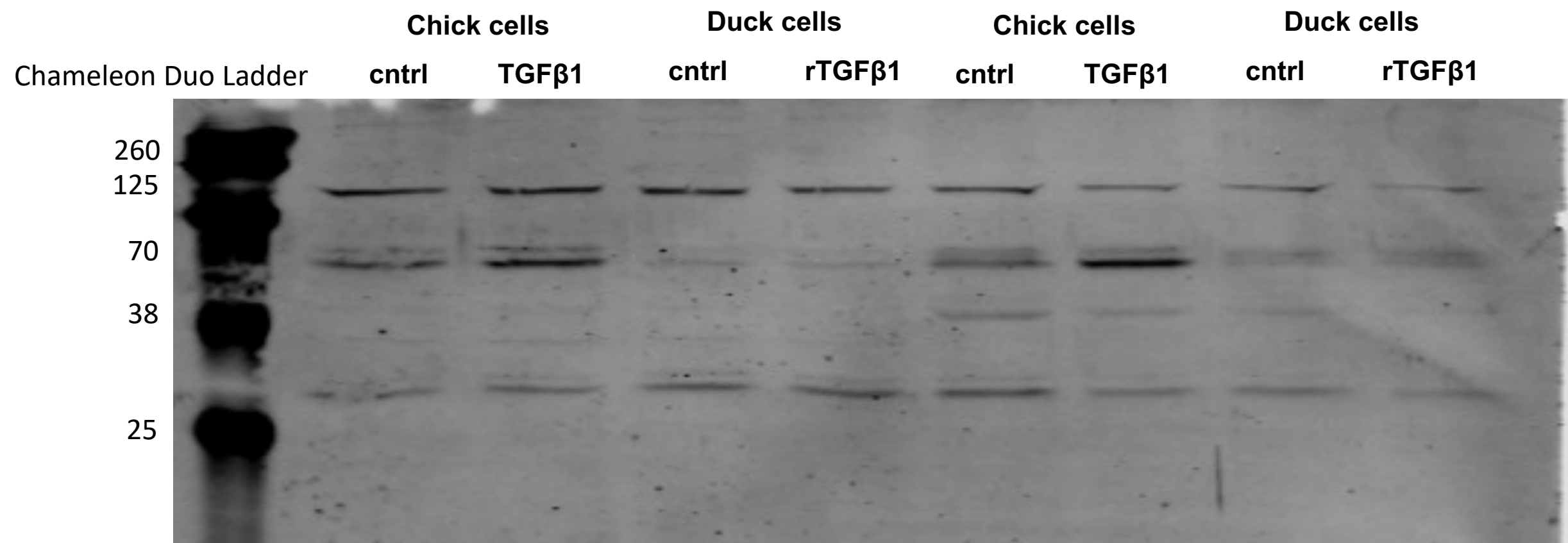

Figure 3-figure supplement 1A-source data 1 ( $\beta$ -Actin)

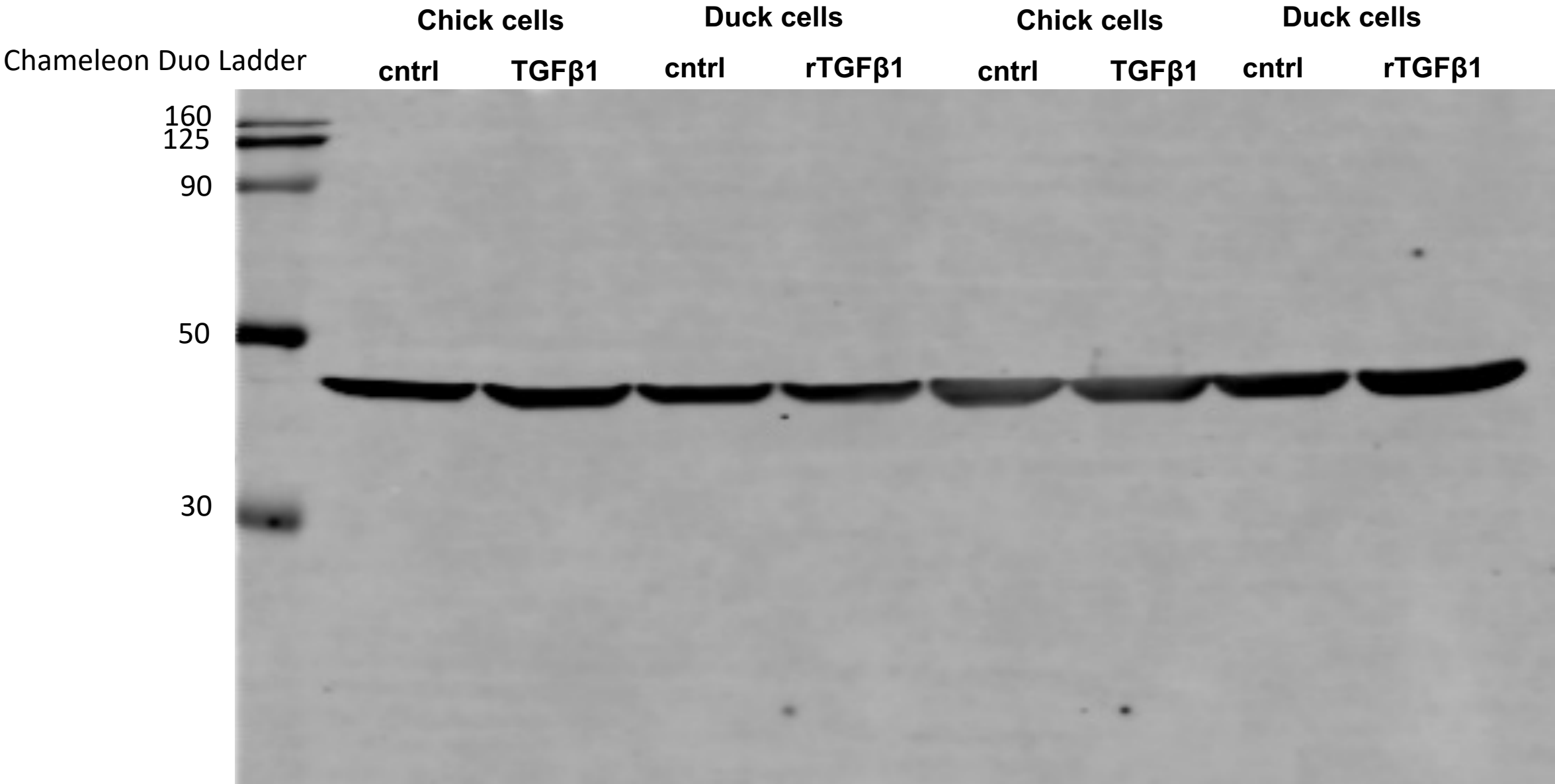

Supplement: Figure 3—figure supplement 1—source data 1. [file elife-66005-fig3-figsupp1-data1.pdf]

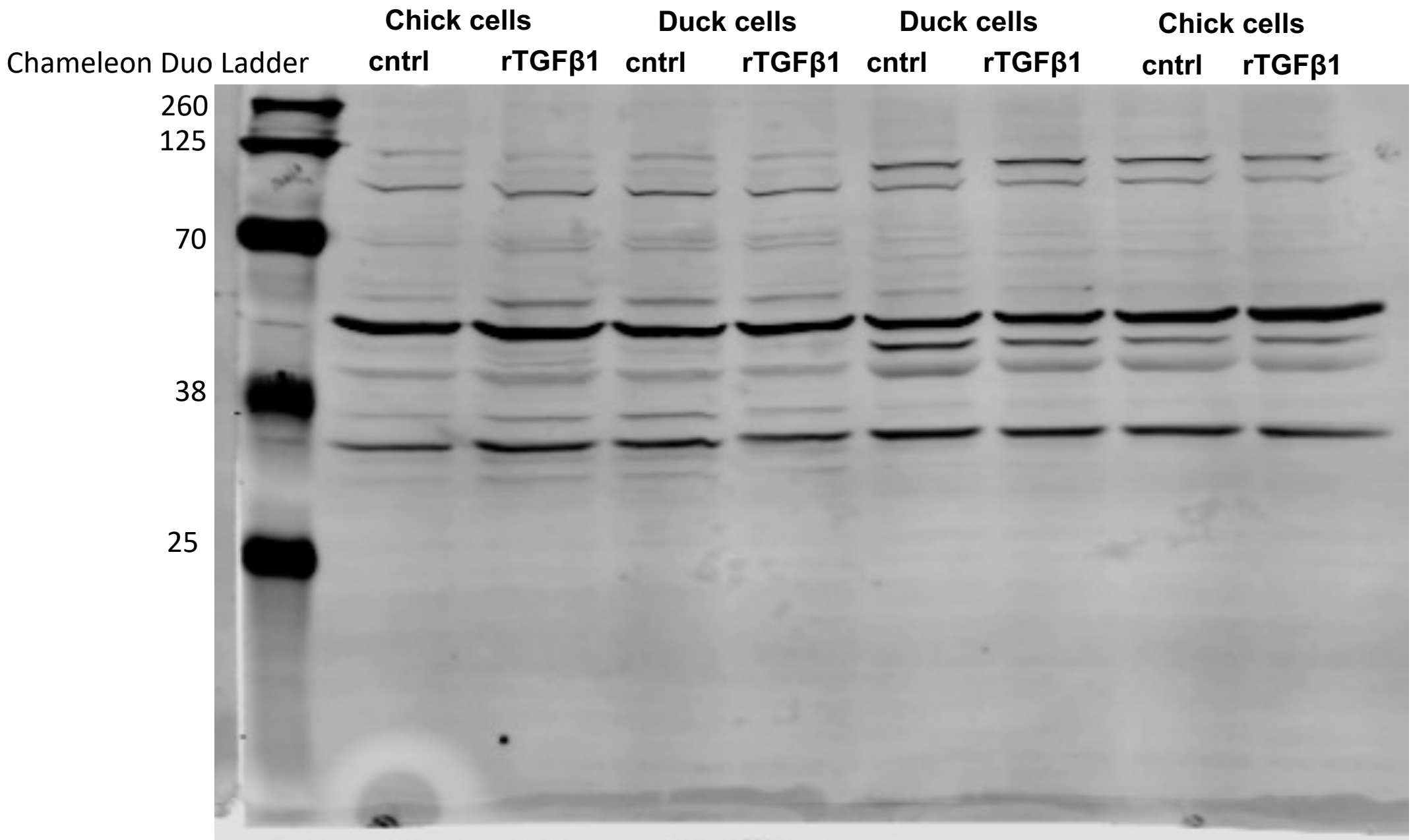

Figure 3-figure supplement 1B-source data 1 ( $\beta$ -Actin)

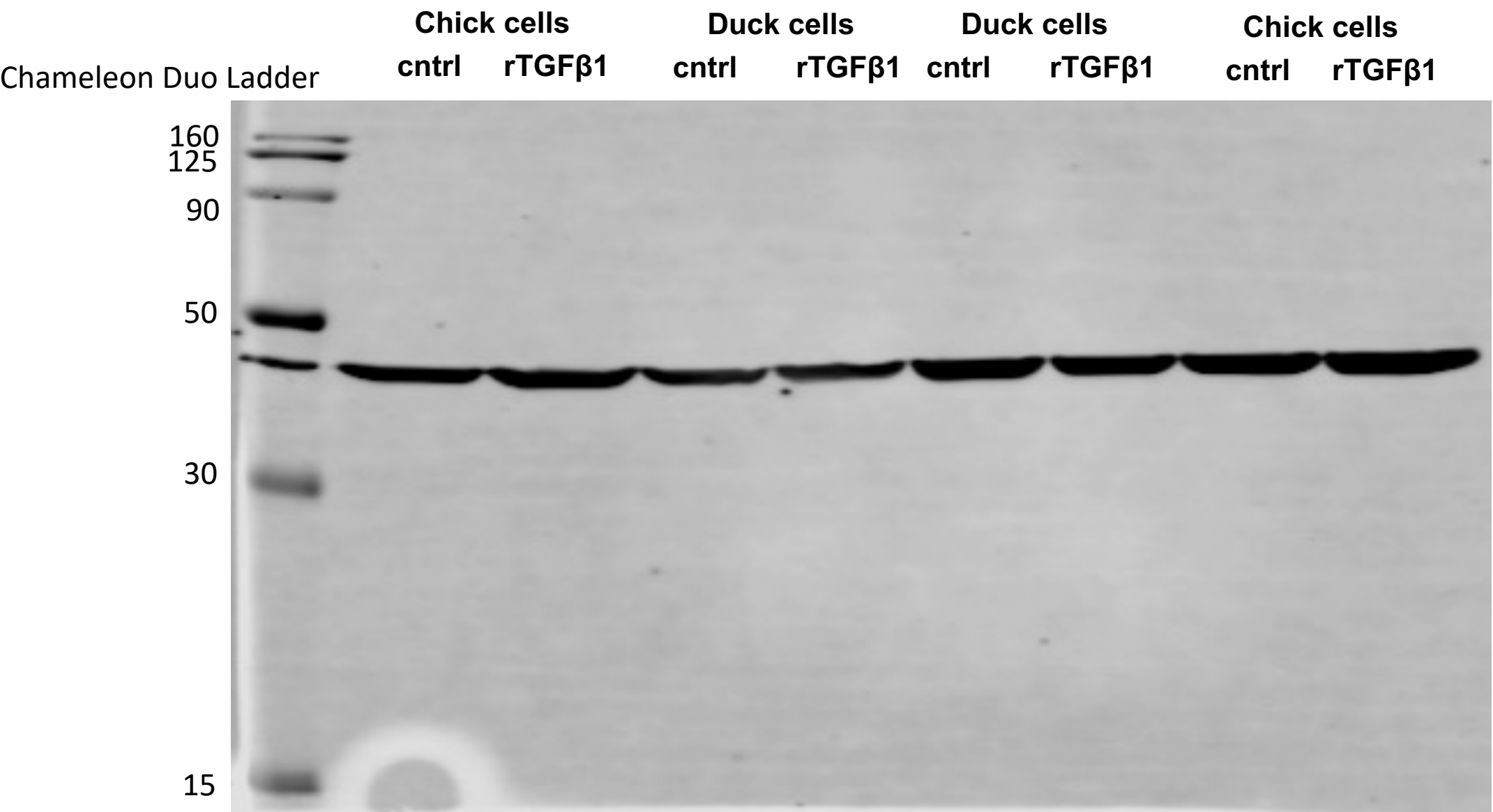

Supplement: Figure 3—figure supplement 1—source data 2. [file elife-66005-fig3-figsupp1-data2.pdf]

quail TGFβi

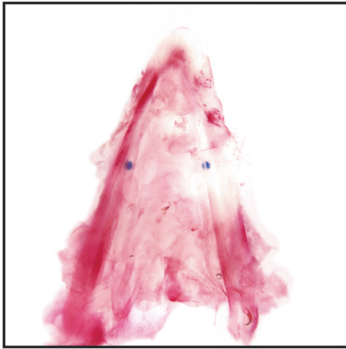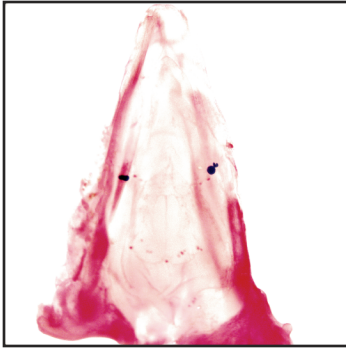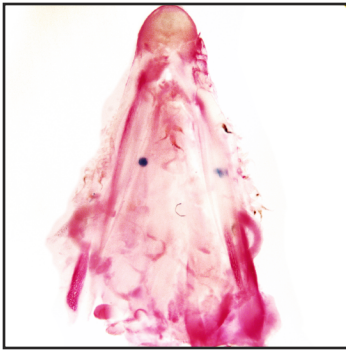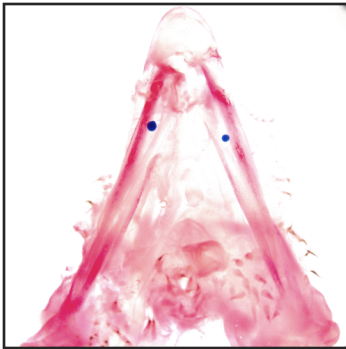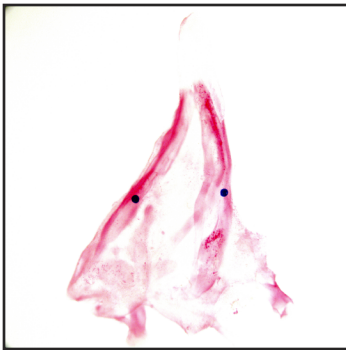

quail SMAD3i

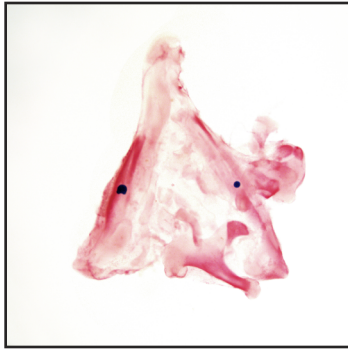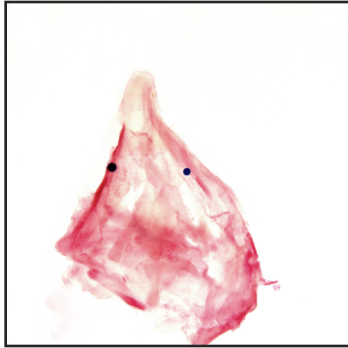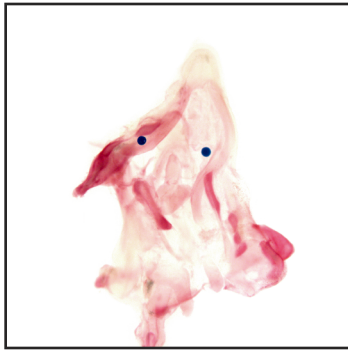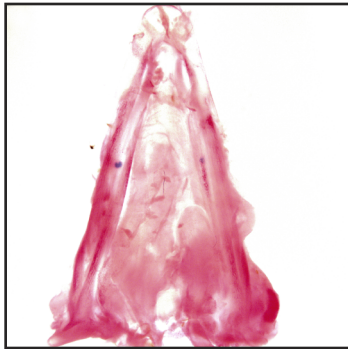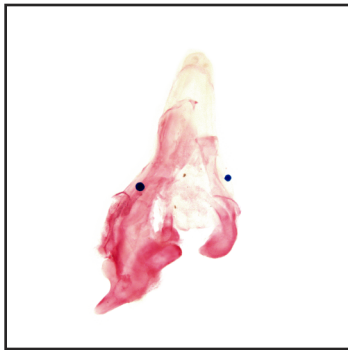

quail MMP13i

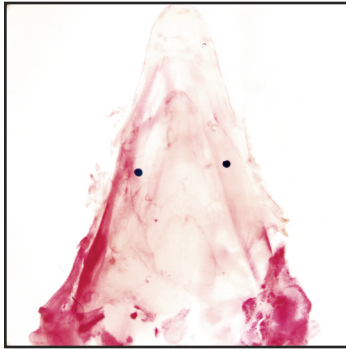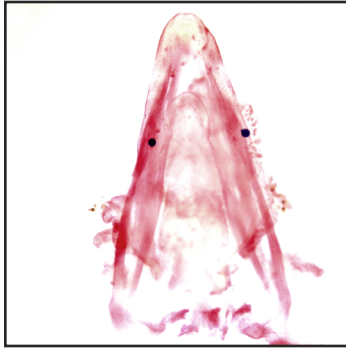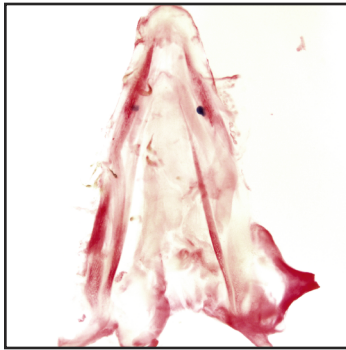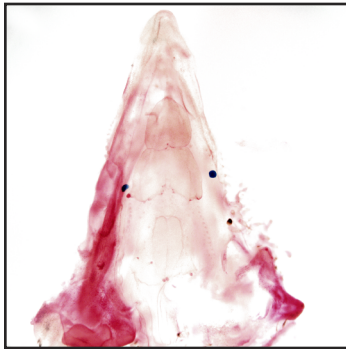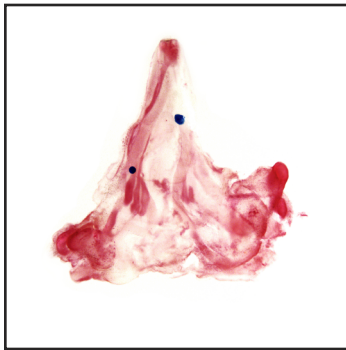

Supplement: Figure 5—source data 1. [file elife-66005-fig5-data1.pdf]

control (empty vector)

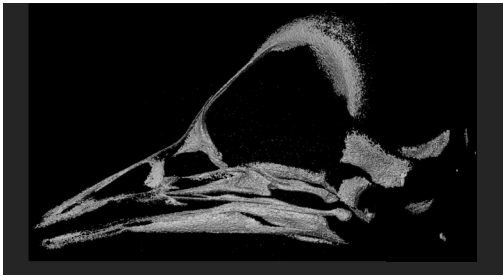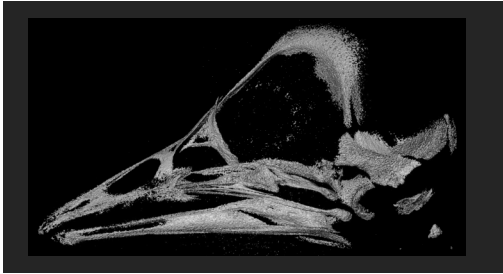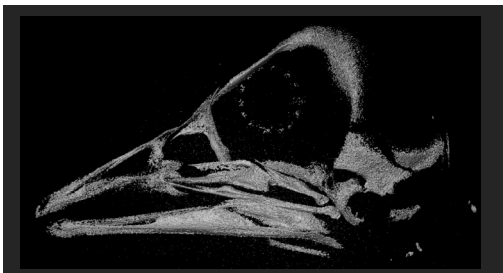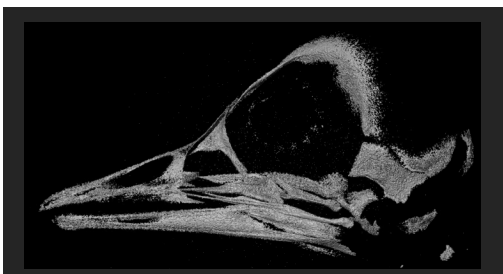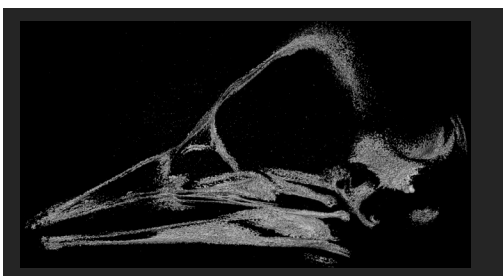

treated (*Mmp13* overexpression)

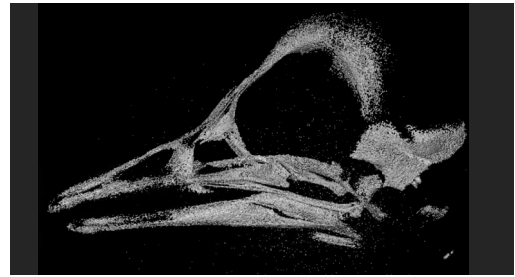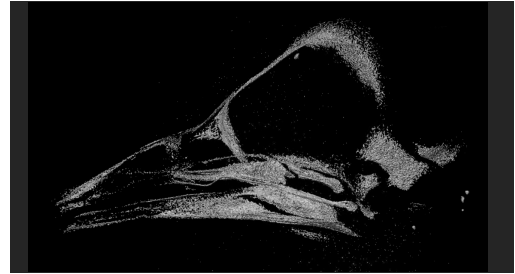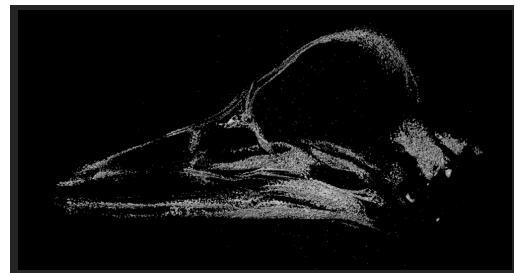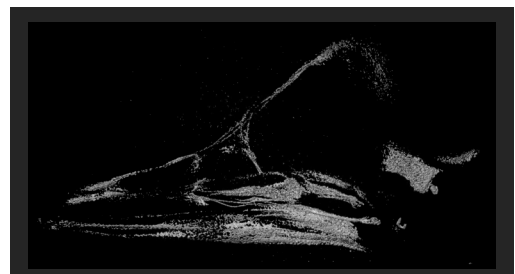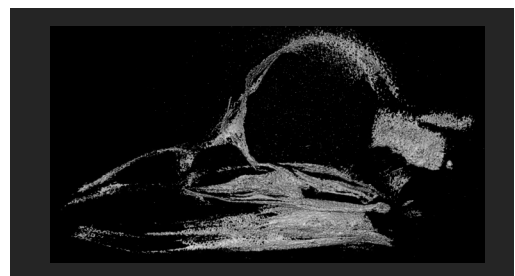

Supplement: Figure 5—source data 2. [file elife-66005-fig5-data2.pdf]
