# Supplementary material for: Species-specific sensitivity to TGFβ signaling and changes to the Mmp13 promoter underlie avian jaw development and evolution
Source: eLife. 2022 Jun 6;11:e66005. doi: 10.7554/eLife.66005 (PMC9246370; doi:10.7554/eLife.66005)

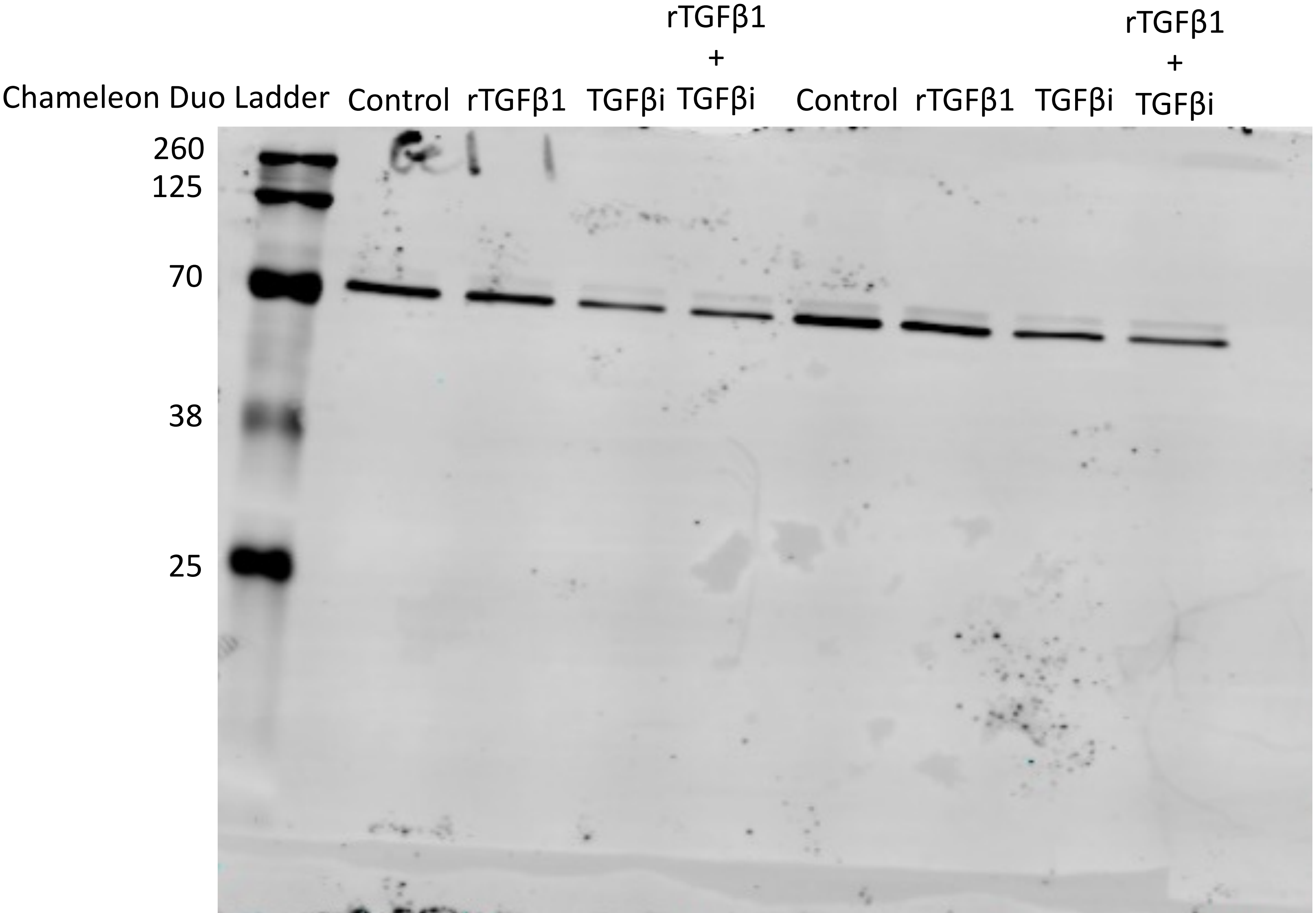
Chameleon Duo Ladder
Control
rTGFβ1
TGFβi
rTGFβ1 + TGFβi
Control
rTGFβ1
TGFβi
rTGFβ1 + TGFβi
260
125
70
38
25

Figure 3-figure supplement 3B-source data 1 (β-Actin)

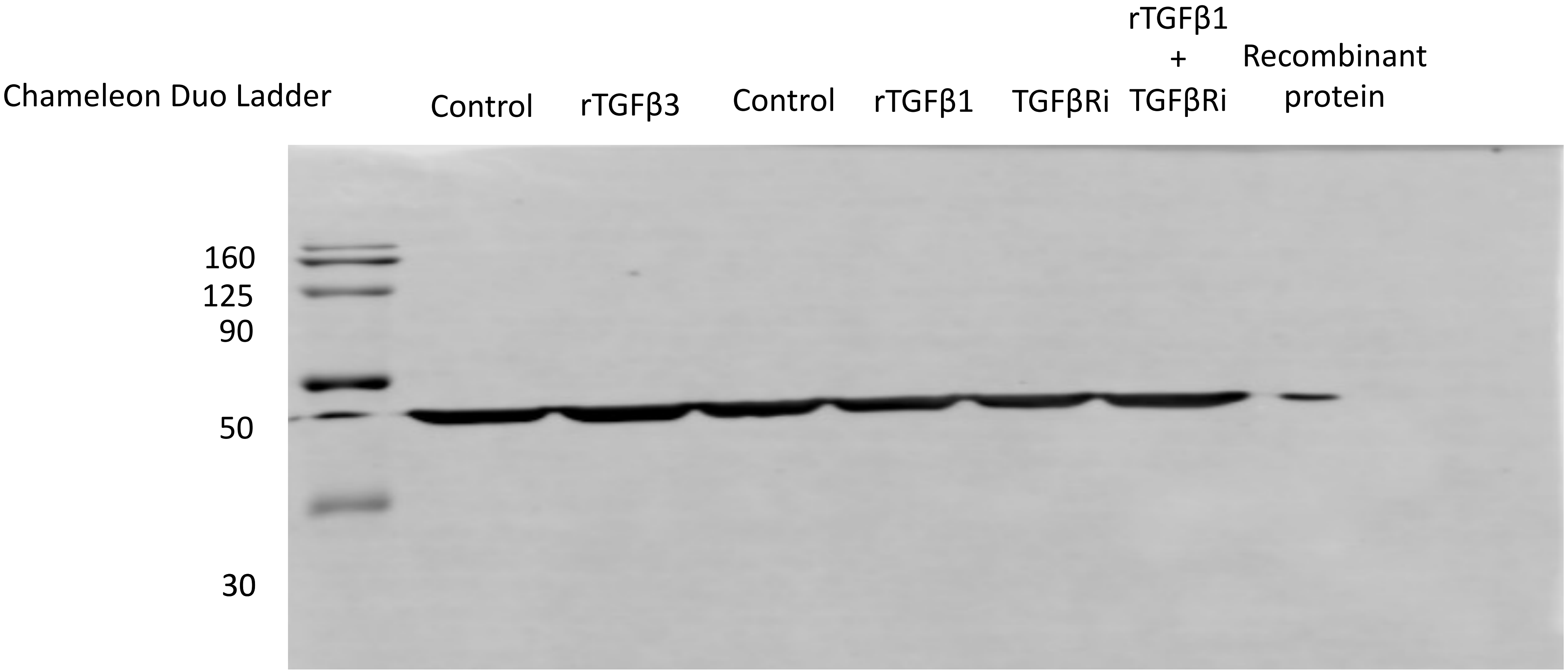

Figure 3-figure supplement 3B-source data 1 (MMP13)

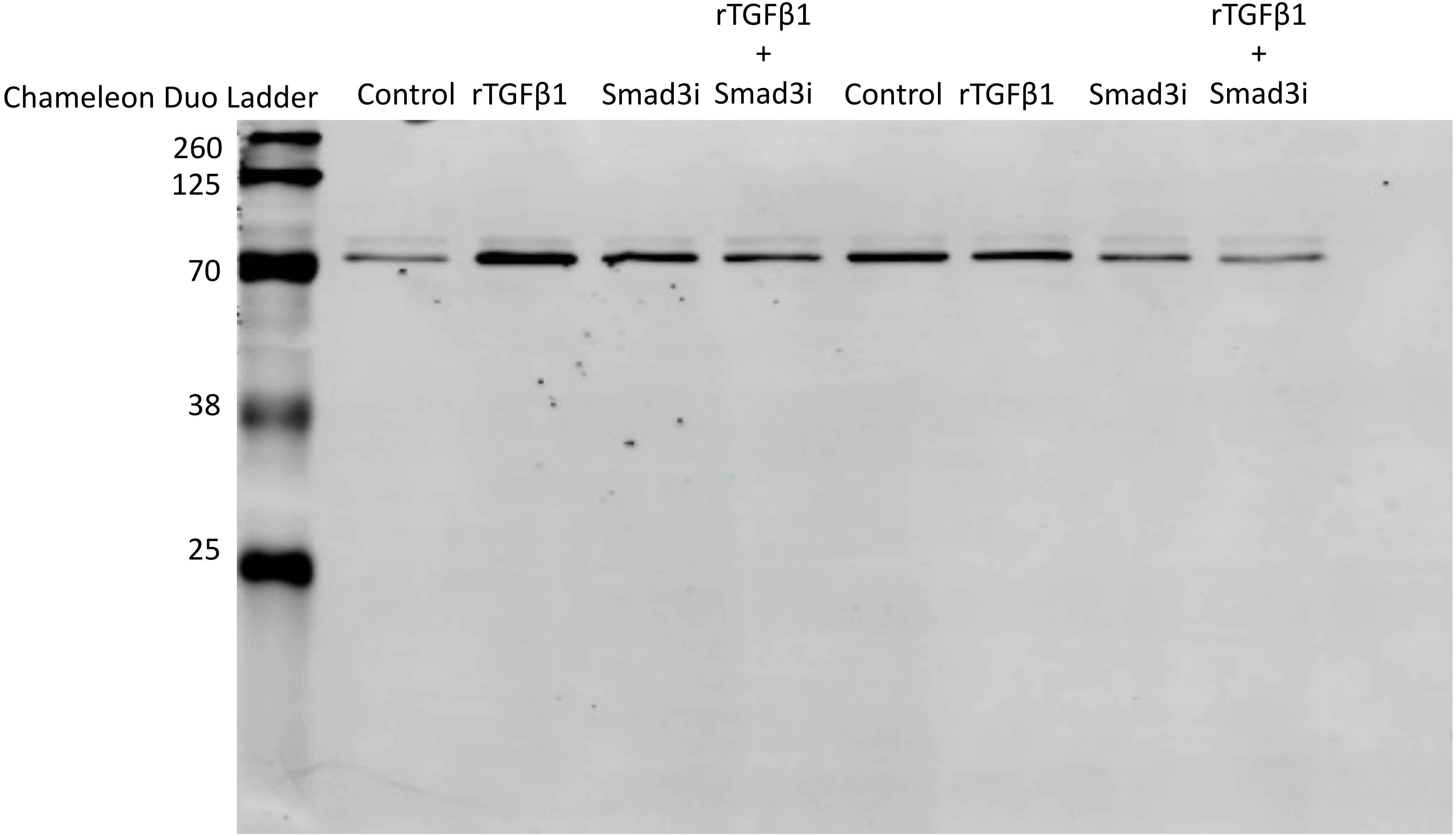

Figure 3-figure supplement 3B-source data 1 (β-Actin)

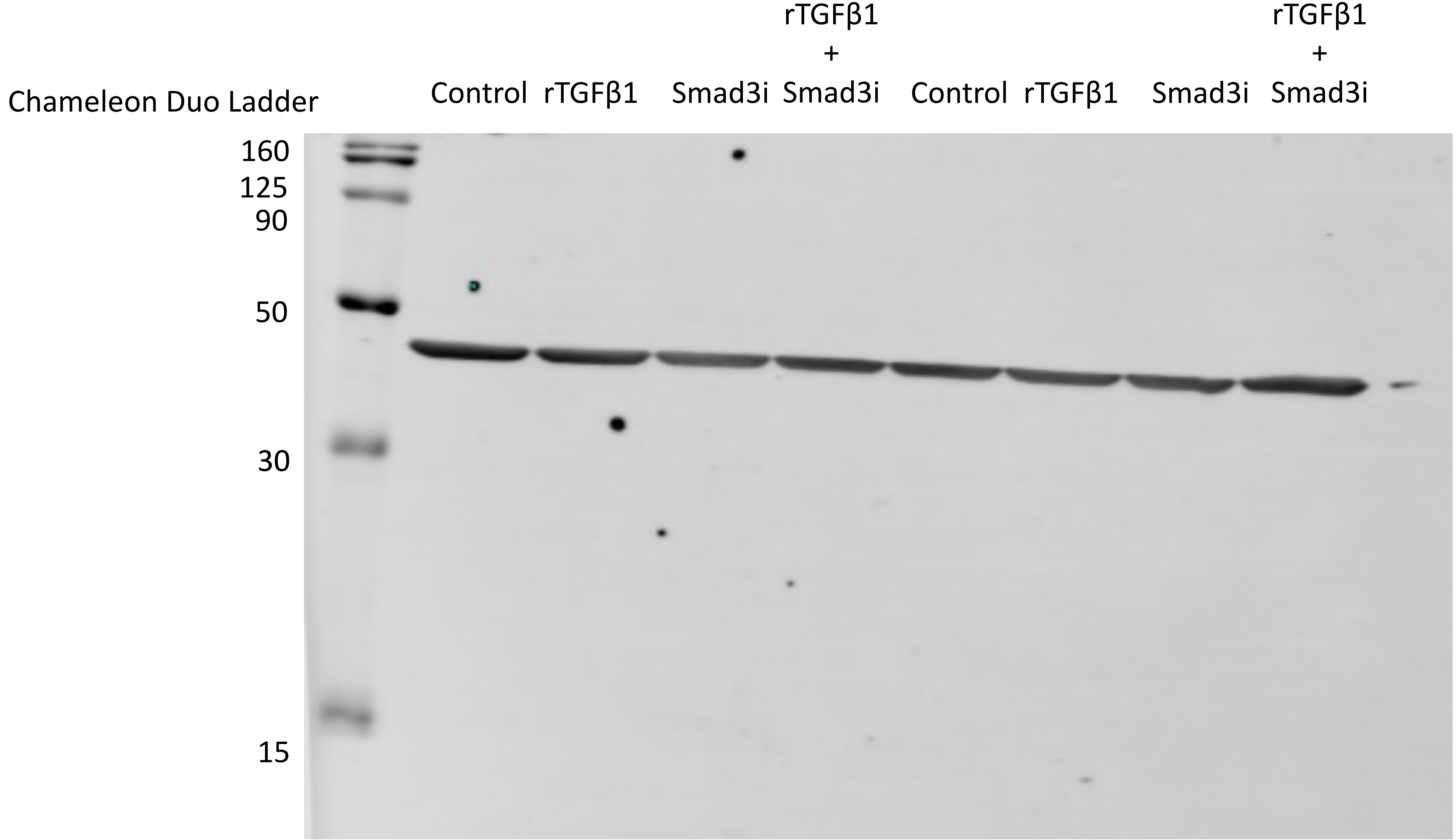

Supplement: Figure 3—figure supplement 3—source data 2. [file elife-66005-fig3-figsupp3-data2.pdf]
