# Supplementary material for: Species-specific sensitivity to TGFβ signaling and changes to the Mmp13 promoter underlie avian jaw development and evolution
Source: eLife. 2022 Jun 6;11:e66005. doi: 10.7554/eLife.66005 (PMC9246370; doi:10.7554/eLife.66005)

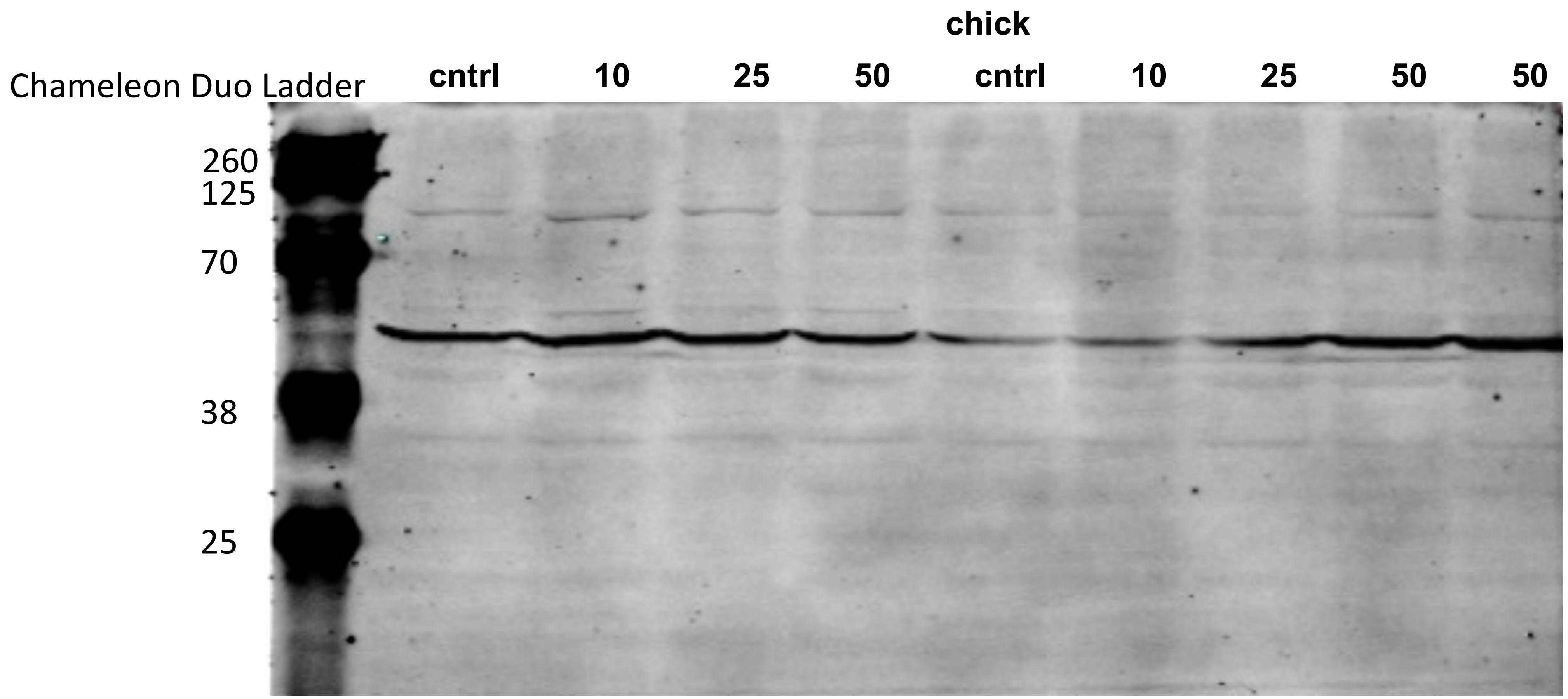
chick
Chameleon Duo Ladder
cntrl
10
25
50
cntrl
10
25
50
50
260
125
70
38
25

Figure 4-figure supplement 1B-source data 1 (β-Actin)

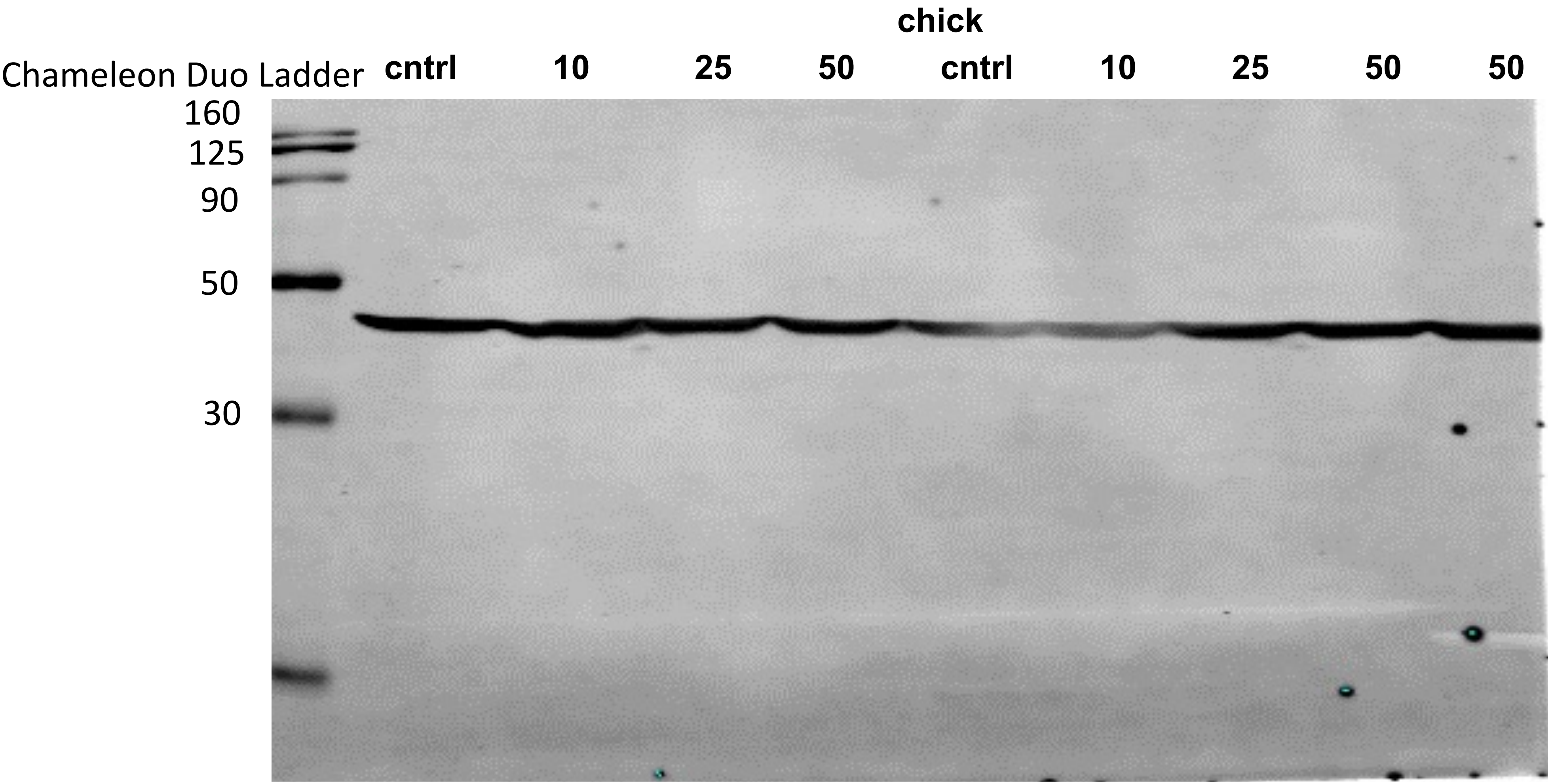

Figure 4-figure supplement 1B-source data 1 (MMP13)

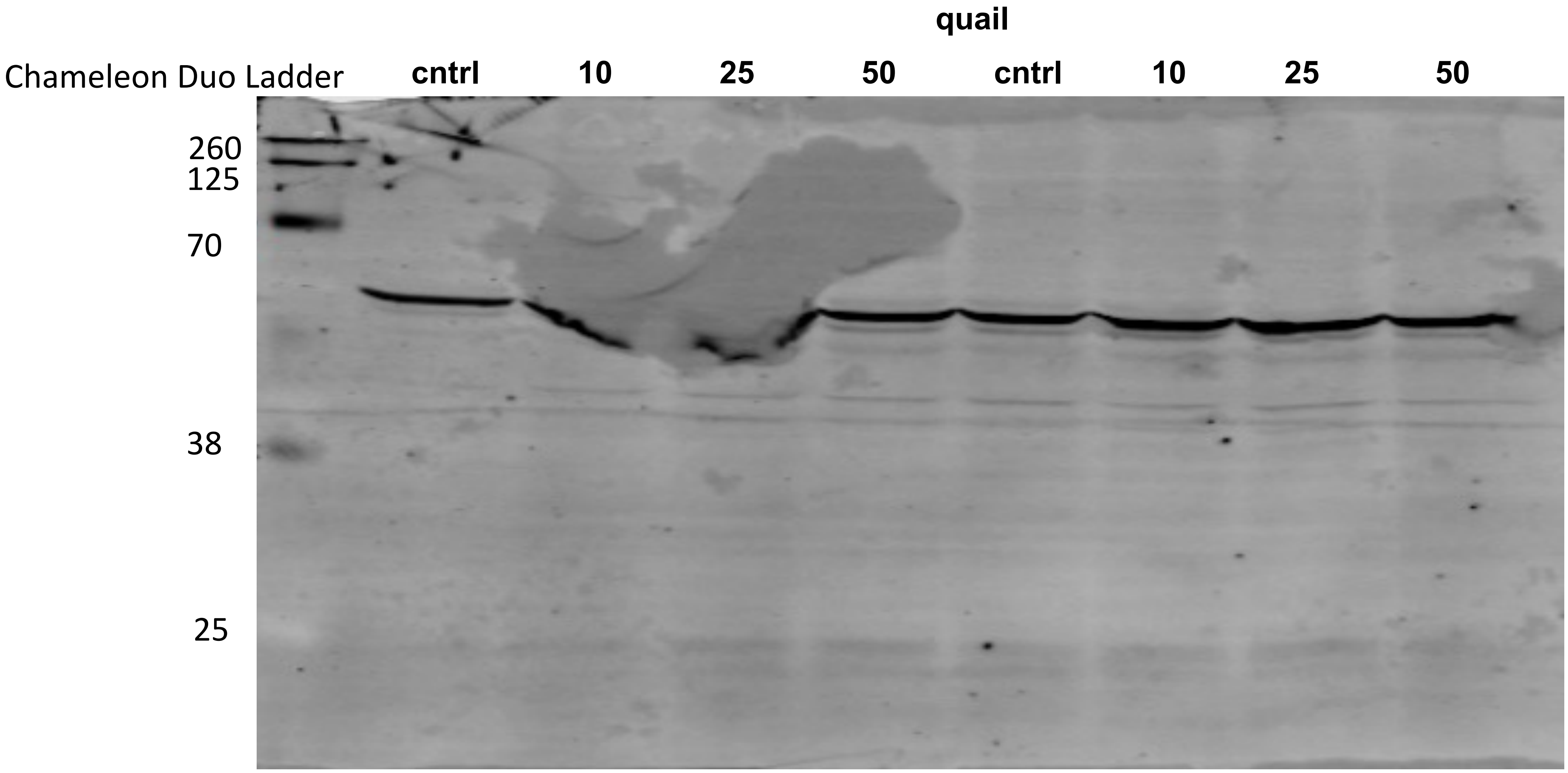

Figure 4-figure supplement 1B-source data 1 (β-Actin)

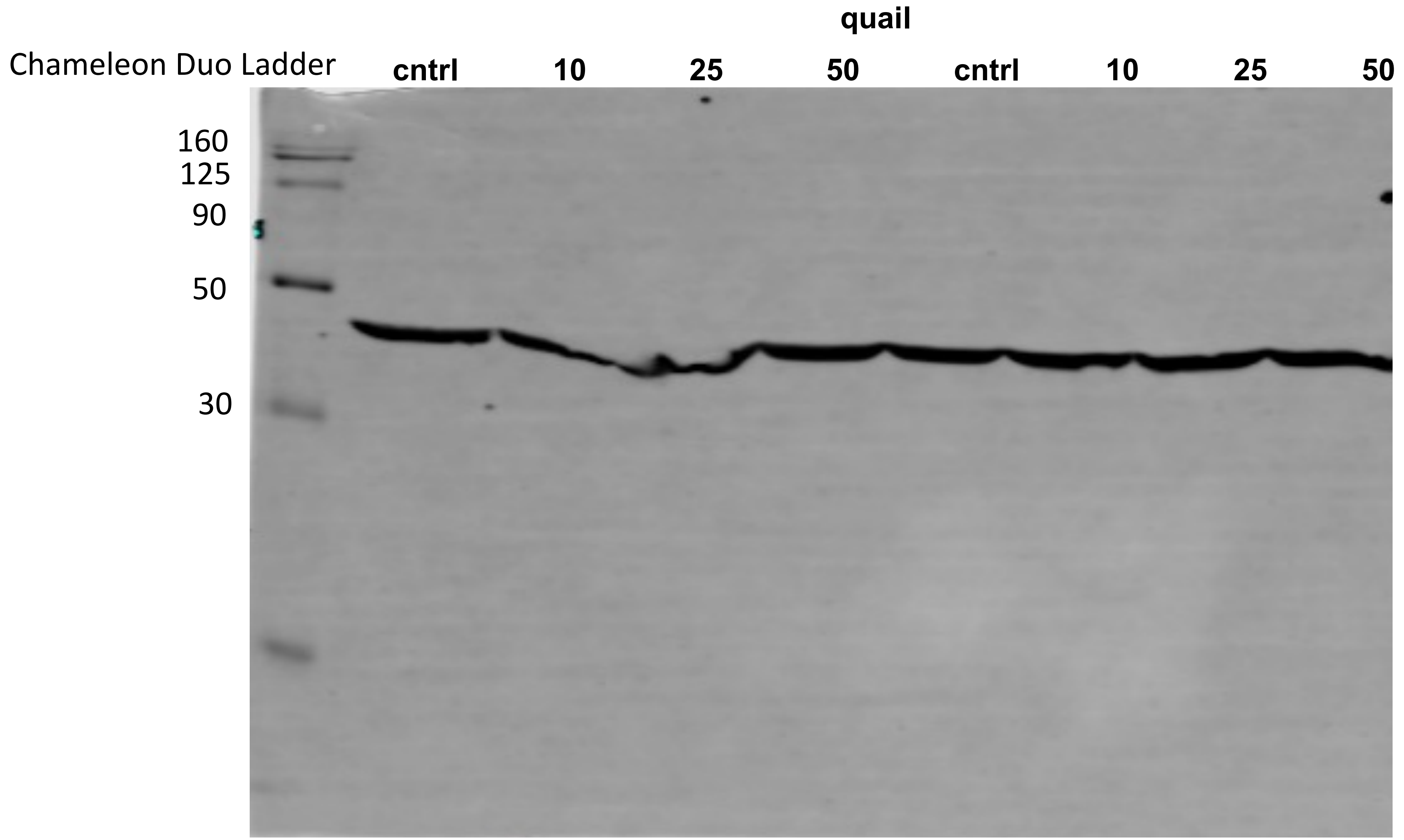

Figure 4-figure supplement 1B-source data 1 (MMP13)

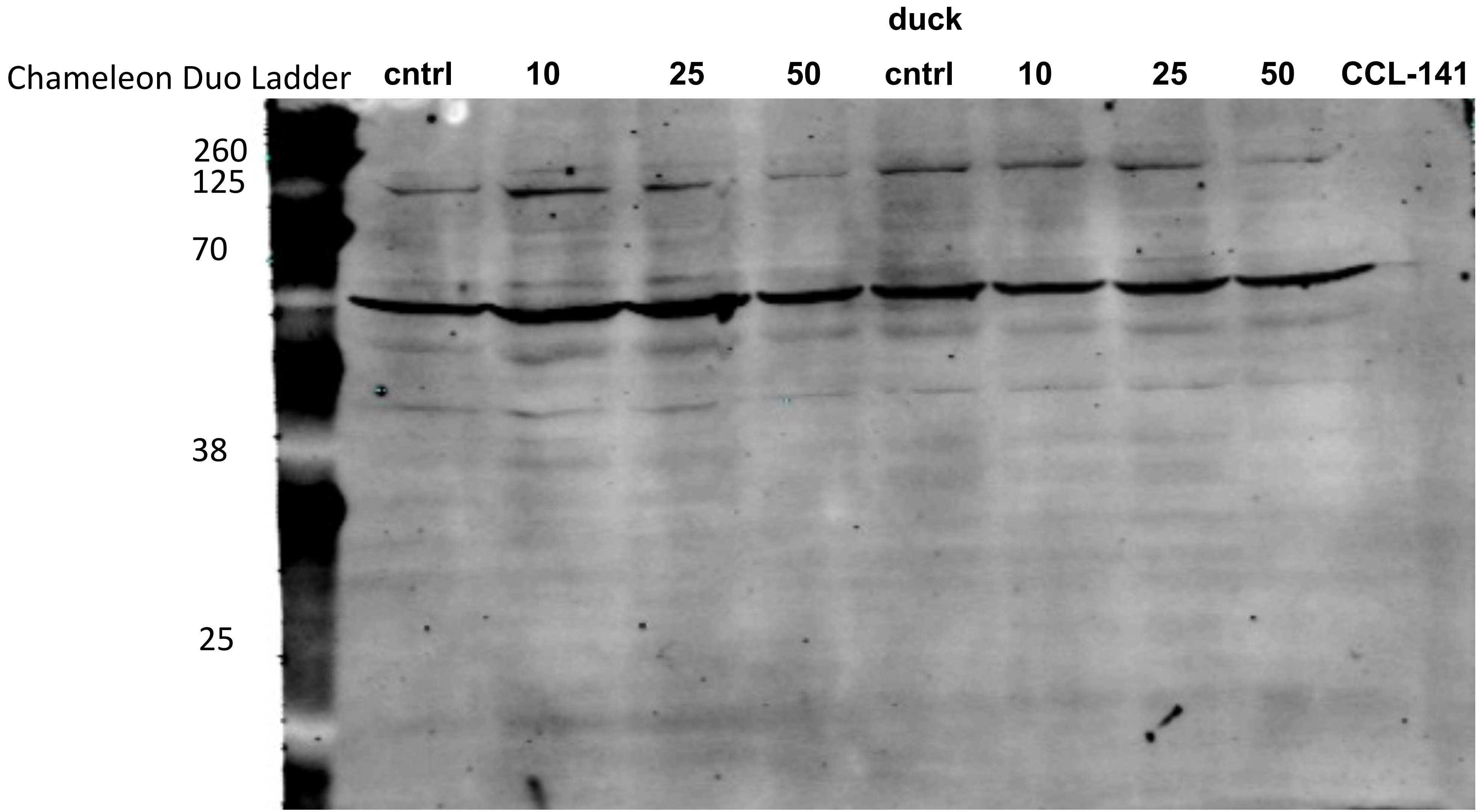

Figure 4-figure supplement 1B-source data 1 (β-Actin)

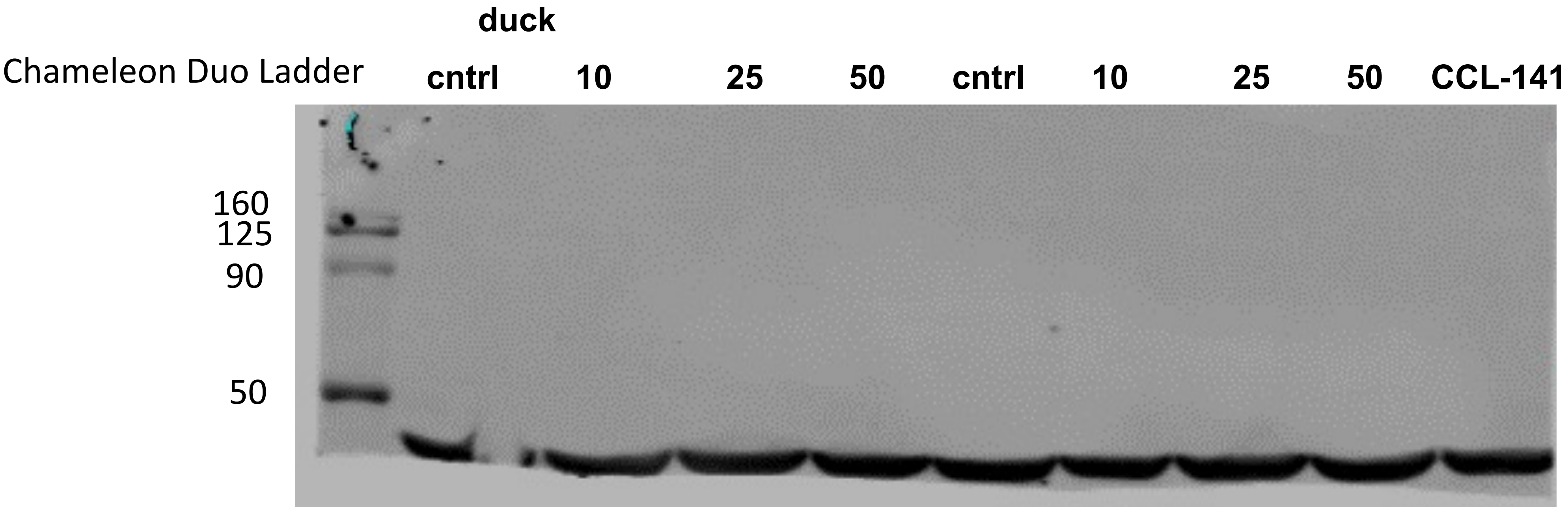

Supplement: Figure 4—figure supplement 1—source data 2. [file elife-66005-fig4-figsupp1-data2.pdf]
