# Supplementary material for: Species-specific sensitivity to TGFβ signaling and changes to the Mmp13 promoter underlie avian jaw development and evolution
Source: eLife. 2022 Jun 6;11:e66005. doi: 10.7554/eLife.66005 (PMC9246370; doi:10.7554/eLife.66005)
Supplement: Supplementary file 1. [file elife-66005-supp1.docx]

| **Reagent type (species) or resource** | **Designation** | **Source or reference** | **Identifiers** |
| --- | --- | --- | --- |
| Eggs (*G. gallus, C. japonica, A. platyrhynchos*) | Chick, quail, duck | AA Labs |  |
| Cell line (*G. gallus*) | DF-1 | ATCC | CRL-12203 |
| Cell line (*A. platyrhynchos*) | CCL-141 | ATCC | CCL-141 |
| Transfected construct (*G. gallus*) | *Mmp13* 2 kb promoter | This paper |  |
| Transfected construct (*C. japonica*) | *Mmp13* 2 kb promoter | This paper |  |
| Transfected construct (*A. platyrhynchos*) | *Mmp13* 2 kb promoter | This paper |  |
| Transfected construct (*G. gallus*) | *Mmp13* -181 bp kb promoter | This paper |  |
| Transfected construct (*C. japonica*) | *Mmp13* -181 bp promoter | This paper |  |
| Transfected construct (*A. platyrhynchos*) | *Mmp13* -181 bp promoter | This paper |  |
| Transfected construct | pPIDNB Empty Vector | Chu et al. 2020  (<https://www.addgene.org/169901/>) |  |
| Transfected construct (*G. gallus*) | pPIDNB *Runx2* overexpression construct | This paper |  |
| Transfected construct (*C. japonica*) | pPIDNB *Runx2* overexpression construct | This paper |  |
| Transfected construct (*A. platyrhynchos*) | pPIDNB *Runx2* overexpression construct | This paper |  |
| Transfected construct (*G. gallus*) | pPIDNB *Mmp13* overexpression construct | This paper |  |
| Transfected construct (*C. japonica*) | pPIDNB M*mp13* overexpression construct | This paper |  |
| Transfected construct (*A. platyrhynchos*) | pPIDNB *Mmp13* overexpression construct | This paper |  |
| Recombinant protein (*H. sapien*) | TGFβ1 | PeproTech | 100-21 |
| Recombinant protein (*H. sapien*) | rMMP13 | MilliporeSigma | 4442875 |
| Recombinant protein (*H. sapien*) | SMAD4 | Abcam | ab81764 |
| Antibody | MMP13 | GenScript | Custom |
| Antibody | Alexa-Fluor 647 | Thermo Fisher Scientific | A32733 |
| Antibody | pSer423/pSer425 SMAD 3 | Novus Biologicals | NBP1-77836 |
| Antibody | MMP2 | Novus Biologicals | NB200-193 |
| Antibody | β-Actin | Novus Biologicals | NB600-501 |
| Antibody | IRDye 800CW | LI-COR | 925-32211 |
| Antibody | IRDye 680RD | LI-COR | 925-68072 |
| Dye | Hoechst 33342 | Thermo Fisher Scientific | 62249 |
| Dye | Opal dye 570 | Akoya Biosciences | FP1488001KT |
| Dye | Opal dye 620 | Akoya Biosciences | FP1495001KT |
| Dye | Opal dye 690 | Akoya Biosciences | FP1497001KT |
| Chemical compound | Paraformaldehyde | Electron Microscopy Sciences | 15714 |
| Chemical compound | Fast Red Violet | MilliporeSigma | F3381 |
| Chemical compound | Ethidium bromide | Bio-Rad | 1610433 |
| Chemical compound | Glycerol | Thermo Fisher Scientific | 525342C |
| Chemical compound | β-mercaptoethanol | MilliporeSigma | M3148-100ml |
| Chemical compound | Reagent Dx | MilliporeSigma | 19088 |
| Chemical compound | RIPA Lysis buffer | MilliporeSigma | 20-188 |
| Chemical compound | Halt protease inhibitors | Thermo Fisher Scientific | 78430 |
| Chemical compound | SB431542 | MilliporeSigma | S4317 |
| Chemical compound | SIS3 | Tocris | 5291 |
| Chemical compound | MMP13 inhibitor | MilliporeSigma | 444283 |
| Chemical compound | Ascorbic acid | Thermo Fisher Scientific | A61-25 |
| Chemical compound | β-Glycerol phosphate | Thermo Fisher Scientific | AC410991000 |
| Chemical compound | Doxycycline | Acros Organics | 446060250 |
| Chemical compound | Luciferase Lysis Buffer | Promega | E1531 |
| Chemical compound | Beetle Luciferin | Promega | E1602 |
| Chemical compound | Galacto-Star β-Galactosidase Reporter | Invitrogen | T1012 |
| Commercial kit | Acid Phosphatase Leukocyte Kit | MilliporeSigma | 387A-1KT |
| Commercial kit | RNAscope Multiplex Fluorescent Reagent Kit v2 | ACD Bio | 323110 |
| Commercial kit | RNAscope Target Retrieval Reagents | ACD Bio | 322000 |
| Commercial kit | RNAscope Multiplex Fluorescent Detection Reagents | ACD Bio | 323110 |
| Commercial kit | RNeasy Plus Mini Kit | Qiagen | 74136 |
| Commercial kit | TURBO DNA-free kit | Invitrogen | AM1907 |
| Commercial kit | iSCRIPT cDNA kit | Bio-Rad | 1708841 |
| Commercial kit | iQ SYBR Green Supermix | Bio-Rad | 1708882 |
| Commercial kit | BCA Assay | Thermo Fisher Scientific | 23225 |
| Commercial kit | NEB Next Single Cell/Low Input cDNA Synthesis Amplification Module | NEB | E6421 |
| Commercial kit | PCR barcoding expansion 1-96 kit | Oxford Nanopore Technologies | EXP-PBC096 |
| Commercial kit | Ligation sequencing kit | ONT | SQK-LSK110 |
| Commercial kit | Purelink Genomic DNA mini | Invitrogen | K1820-01 |
| Commercial kit | GeneJET PCR Purification Kit | Thermo Fisher Scientific | K0702 |
| Commercial kit | Rapid DNA Ligation Kit | Thermo Fisher Scientific | K1422 |
| Commercial kit | NEBuilder HiFi DNA Assembly Master Mix | NEB | E2321L |
| Commercial kit | Purelink Fast Low Endotoxin Midi kit | Invitrogen | A36227 |
| Commercial kit | Maxima H Minus first strand synthesis kit | Thermo Fisher Scientific | K1651 |
| Commercial kit | Lipofectamine 3000 | Invitrogen | L3000008 |
| Commercial kit | Nuclear extraction kit | Abcam | ab113474 |
| Commercial kit | LightShift Chemiluminescent EMSA kit | Thermo Fisher Scientific | 20148 |
| Reagent | DMEM | Corning | 10-013-CV |
| Reagent | MEMα | Thermo Fisher Scientific | A10490-01 |
| Reagent | FBS | VWR | 97068-085, Lot 283K18 |
| Reagent | Penicillin-streptomycin | Thermo Fisher Scientific | 15140122 |
| Enzyme | Protease Plus | ACD Bio | 322381 |
| Enzyme | EcoR1-HF | NEB | R3101S |
| Enzyme | Q5 Hot Start High-Fidelity DNA Polymerase | NEB | M0493L |
| Enzyme | Xba1 | NEB | R0145S |
| Enzyme | HindIII-HF | NEB | R3104S |
| Enzyme | XhoI | NEB | R0146S |
| Enzyme | AFIII | NEB | R0520S |
| Enzyme | PstI | NEB | R3140S |
| Enzyme | Coenzyme A | Thermo Fisher Scientific | J13787MF |
| Equipment | Incubator | GQF Hova-Bator | 1588 |
| Equipment | Nikon AZ100 C2 | Nikon |  |
| Equipment | Leica DM 2500 | Leica Microsystems |  |
| Equipment | SPOT Insight 4 Megapixel CCD | Diagnostic Instruments |  |
| Equipment | Leica MZFLIII | Leica MZFLIII |  |
| Equipment | SP8 confocal microscope | Leica Microsystems |  |
| Equipment | HC PL APO 20x/0,75 IMM CORR CS2 lens | Leica Microsystems | 506343 |
| Equipment | Bead Mill 24 Homogenizer | Thermo Fisher Scientific | 15-340-163 |
| Equipment | SpectraMax M5 microplate reader | Molecular Devices |  |
| Equipment | Odyssey Imaging System | LI-COR |  |
| Equipment | Pneumatic PicoPump | World Precision Instruments | PV830 |
| Equipment | Micropipette puller | Sutter Instrument | P-97 Flaming/Brown |
| Equipment | CUY21EDITII Next Generation Electroporator | BEX CO, Ltd |  |
| Supplies | 1.4 mm ceramic beads | Thermo Fisher Scientific | 15-340-153 |
| Supplies | 2.8 mm ceramic beads | Thermo Fisher Scientific | 15-340-154 |
| Supplies | Immobilon-P PVDF membrane | MilliporeSigma | IPVH00010 |
| Supplies | 0.45 μm membrane filter | MilliporeSigma | HAWP01300 |
| Supplies | 6-well transwell inserts | VWR | 10769-192 |
| Supplies | Affigel Blue Beads | Bio-Rad | 1537301 |
| Supplies | 24 well plates | Corning | 353047 |
| Supplies | Nylon membrane | Roche | 11209272001 |
| Supplies | Borosilicate glass micropipettes (O.D. 1.0 mm, I.D. 0.75 mm) | Sutter Instrument | B100-75-10 |
| Software | Geneious Prime | Geneious | Version 2020.2.4 |
| Software | Dragonfly | Object Research Systems | v.4.1 |
| Software | Prism | Graphpad | Version 9.0.0 |
| Software | Adobe Illustrator | Adobe | Version 24.2.3 |
| Software | Dragonfly v.4.1.0.647 | Object Research Systems |  |
