# Supplementary material for: Species-specific sensitivity to TGFβ signaling and changes to the Mmp13 promoter underlie avian jaw development and evolution
Source: eLife. 2022 Jun 6;11:e66005. doi: 10.7554/eLife.66005 (PMC9246370; doi:10.7554/eLife.66005)
Supplement: Supplementary file 2. [file elife-66005-supp2.docx]

|  | Antigen Sequence |
| --- | --- |
| MMP13 antibody for chick, quail, duck. Affinity-purified peptide supplied polyclonal. Host strain: New Zealand rabbit. | MHHHHHHAPLHSKPQAVITFPGELLSAPSDVELAENY  LLRFGYIQEAEVRRSSKHVSLAKALRRMQKQLGLEET  GELDASTLEAMRAPRCGVPDVGGFLTFEDELKWDHM  DLTYRVMNYSPDLDRAVIDDAFRRAFKVWSDVTPLTF  TQIYSGEADIMIMFGSQEHGDGYPFDGKDGLLAHAFPP  GSGIQGDAHFDDDEFWTLGTGLEVKTRYGNANGASCH  FPFIFEGRSYSRCITEGRTDGMLWCATTASYDADKTYGF  CPSELLYTNGGNSDGSPCVFPFIFDGASYDTCTTDGRSD  GYRWCATTANFDQDKKYGFCPNRDTAAIGGNSQGDPC  VFPFTFLGQSYSARTSQGRQDGKLWCATTSNYDTDKK  WGFCPDPGYSIFLVAAH |
