# Supplementary material for: Species-specific sensitivity to TGFβ signaling and changes to the Mmp13 promoter underlie avian jaw development and evolution
Source: eLife. 2022 Jun 6;11:e66005. doi: 10.7554/eLife.66005 (PMC9246370; doi:10.7554/eLife.66005)
Supplement: Supplementary file 3. [file elife-66005-supp3.docx]

| Gene | Sequence |
| --- | --- |
| Chick/quail/duck *18S* (sense)  Chick/quail/duck *18S* (antisense) | 5’- GCGTGTGCCTACCCTACGCC -3’  5’- ACGCAAGCTTATGGCCCGCA -3’ |
| Chick/quail/duck *Tgfβ1* (sense)  Chick/quail/duck *Tgfβ1* (antisense) | 5’- TCATGACATGAACCGGCC -3’  5’- AGTCAATGTACAGCTGCC -3’ |
| Chick/quail/duck *Tgfβ3* (sense)  Chick/quail/duck *Tgfβ3* (antisense) | 5’- AATGCACTGCTATCTCCTG -3’  5’- CTTCAGCTTGCTCAGGATC -3’ |
| Chick/quail/duck *Tgfβr1* (sense)  Chick/quail/duck *Tgfβr1* (antisense) | 5’- GTACATGGCACCTGAAGTTC -3’  5’- GGCAACTGGTAATCTTCATG -3’ |
| Chick/quail/duck *Smad2* (sense)  Chick/quail/duck *Smad2* (antisense) | 5’- TTGCTGCTCTTCTGGCTC -3’  5’- TGAAGTTCAATCCAGCAAGG -3’ |
| Chick/quail *Runx2* *full length* (sense)  Duck *Runx2* *full length* (sense)  Chick/quail/duck *Runx2 full length* (antisense) | 5’- GCTGTGATGAAGAACCAGGT -3’  5’- GCCGTGATGAGGAACCAGGT -3’  5’- TGGTATGTAGCGACTTGGGG -3’ |
| Chick/quail/duck *Mmp13* (sense)  Chick/quail/duck *Mmp13* (antisense) | 5’- TTGGCTTGGAGGTGACAG -3’  5’- GAACATCAGTGCTCCAGG -3’ |
| Chick/quail/duck *Tgfβ2* (sense)  Chick/quail/duck *Tgfβ2* (antisense) | 5’- AATGCACTGCTATCTCCTG -3’  5’- CTTCAGCTTGCTCAGGATC -3’ |
| Chick/quail/duck *Acvrl1* (sense)  Chick/quail/duck *Acvrl1* (antisense) | 5’- GTGAGACCGAGATCTACAAC -3’  5’- TCTGCAGGTAGTCGTAGAG -3’ |
| Chick/quail/duck *Tgfβr2* (sense)  Chick/quail/duck *Tgfβr2* (antisense) | 5’- CTCACAAGAAGAGGAAGCTC -3’  5’- CTGTGTTGTGGTTGATGTTG -3’ |
| Chick/quail/duck *Tgfβr3* (sense)  Chick/quail/duck *Tgfβr3* (antisense) | 5’- ATGTGGATATGATCTTGGCC -3’  5’- AGAGTGTCCAGGCCATAG -3’ |
| Chick/quail/duck *Smad3* (sense)  Chick/quail/duck *Smad3* (antisense) | 5’- CCTATGTCTCCAGCACAC -3’  5’- AAGCCATCCACGGTCATG -3’ |
| Chick/quail/duck *Pai1* (sense)  Chick/quail/duck *Pai1* (antisense) | 5’- TAGTGGCAGCGATAGATCC -3’  5’- ATCAGCAGGCGGATGTTC -3’ |
| Chick/quail/duck *Mmp2* (sense)  Chick/quail/duck *Mmp2* (antisense) | 5’- GATGATGACCGCAAGTGG -3’  5’- GGAAGTTCTTGGTGTAGGTG -3’ |
| Chick/quail/duck *Mmp9* (sense)  Chick/quail/duck *Mmp9* (antisense) | 5’- GACACCGACAAGAAGTGG -3’  5’- GAAGTCCTGGACGTAGCTG -3’ |
| Chick *Runx2* *promoter 1* (sense)  Quail/duck *Runx2* *promoter 1* (sense)  Chick/quail *Runx2 promoter 1 (*antisense)  Duck *Runx2* *promoter 1* (antisense) | 5’- CGACAGGAACTATGGCATCA -3’  5’- CGAGAGGGACTATGGCATCA -3’  5’- CTGTTGGGCCACTACGACC -3’  5’- CTGTTGGGCTACTACGACC -3’ |
| Chick/quail/duck *Runx2 promoter 2* (sense)  Chick/quail *Runx2 promoter 2 (*antisense)  Duck *Runx2 promoter 2* (antisense) | 5′- TTGTGGCTGTTGTGATGCG -3′  5′- CTGTTGGGCCACTACGACC -3′  5’- CTGTTGGGCTACTACGACC -3’ |
| Chick/quail/duck *Runx2 exon 5* (sense)  Chick/quail/duck *Runx2 exon 5 (*antisense) | 5′- GTCGCTACATACCACAGAGC -3′  5′- TCGGGACCCCTACTCTCATA -3′ |
| Chick/quail/duck *Runx2 exon 4,6* (sense)  Chick *Runx2* *exon 4,6* (antisense)  Quail/duck *Runx2 exon 4,6 (*antisense) | 5′- GTCGCTACATACCACAGAGC -3′  5′- GGAAGTCCTGTGCCTCGG -3′  5′- GGAAGTCCTGTGCCTCGA -3′ |
| Chick/quail/duck *Runx2 c-terminus 1* (sense)  Chick/quail/duck *Runx2 c-terminus 1* (antisense) | 5′- GACTTCCAGCCATCACCGA -3′  5′- AGTGAGTGGTTGCGGACATA -3′ |
| Chick/quail/duck *Runx2 c-terminus 2* (sense)  Chick/quail *Runx2 c-terminus 2* (antisense)  Duck *Runx2* *c-terminus 2* (antisense) | 5′- GACTTCCAGCCATCACCGA -3′  5′- GAAGTTGGTGGAGAAGTGGC -3′  5′- GAAGTTGGTGGTGAAGTGGC -3′ |
| Chick/quail/duck *Ocn (sense)*  Chick/quail/duck *Ocn (antisense)* | 5′- TCACATTCAGCCTCTGCCG -3′  5′- GCTCACACACCTCTCGTTGG -3′ |
| Chick/quail *Col1a1* (sense)  Duck *Col1a1* (sense)  Chick/quail *Col1a1* (antisense)  Duck *Col1a1* (antisense) | 5′- AGAACAGCGTCGCCTACATG -3′  5′- TGCCTTCATCTGGTGCTCAC -3′  5′- CTCCAGTGTGACTCGTGCAG -3′  5′- GTGTCCTGGTCCATGTAGGC -3′ |
| Chick/quail/duck *Alp (sense)*  Chick/quail/duck *Alp* (antisense) | 5′- TCCACCAGCAGGAAGAAG -3′  5′- GGATATGGTGTATGAGCTGG -3′ |
| Chick *Mmp13* *full length* *(sense)*  Chick *Mmp13* *full length* (antisense) | 5′- ATGCAACCCAGACTTTCAGC -3′  5′- GGTAGTCAGTGCTTGTTCGC -3′ |
| Duck *Mmp13* *full length* *(sense)*  Duck *Mmp13* *full length* (antisense) | 5′- TCAGGGCTTCAGACTTCACA -3′  5′- AGCCTGCAACATGTCATTCA -3′ |
| Quail *Mmp13* *full length* *(sense)*  Quail *Mmp13* *full length* (antisense) | 5′- ACACATCAGGGCTTCAGACT -3′  5′- TGCAGCTTGTAACATGGCAC -3′ |
| Chick pTet *Mmp13* *(sense)*  Quail pTet *Mmp13 (sense)*  Chick/quail pTet *Mmp13* (antisense)  Duck pTet *Mmp13 (sense)*  Duck pTet *Mmp13* (antisense) | 5′-ACCCTCGTAAAGCCGCCACCATGCAACCCAGAC  TTTCAGC -3′  5′-ACCCTCGTAAAGCCGCCACCATGCAACCAAGAC  TTTCAGC -3′  5′-GCCGCTTCACTTGTACTGCATCAGCACCAAAAT  AAGGAGT -3′  5′-ACCCTCGTAAAGCCGCCACCATGATGCAGTCAA  GGCTTTC -3′  5′- GCCGCTTCACTTGTACTGCATCAGCACCAAAAT  ­­­­­ATGGAGT -3′ |
