# Supplementary material for: Species-specific sensitivity to TGFβ signaling and changes to the Mmp13 promoter underlie avian jaw development and evolution
Source: eLife. 2022 Jun 6;11:e66005. doi: 10.7554/eLife.66005 (PMC9246370; doi:10.7554/eLife.66005)
Supplement: Supplementary file 4. [file elife-66005-supp4.docx]

| **Chick** | | | | | | |
| --- | --- | --- | --- | --- | --- | --- |
| Transcription factor | Position | Absolute score | Relative score | Strand | JASPAR ID | Sequence |
| SMAD2::SMAD3 | 73-86 | 13.71 | 0.95 | + | MA1622.1 | GTGGTGACTCACTG |
| RUNX2 | 163-171 | 11.84 | 0.96 | + | MA0511.2 | AAACCACAG |
| RUNX2 | 189-197 | 9.40 | 0.93 | + | MA0511.2 | AGACCACAG |
| SMAD4 | 1046-1053 | 8.19 | 0.93 | - | MA1153.1 | AGTCTGGG |
| RUNX2 | 1108-1116 | 9.74 | 0.94 | - | MA0511.2 | AAACCTCAA |
| SMAD2::SMAD3 | 1252-1265 | 11.38 | 0.91 | + | MA1622.1 | TAAATGACTAATAC |
| SMAD3_1 | 1841-1857 | 11.21 | 0.90 | + | PB0060.1 | CTTCTCCAGACTGAACA |
| SMAD4 | 1846-1853 | 10.17 | 0.96 | - | MA1153.1 | AGTCTGGA |
| **Duck** | | | | | | |
| SMAD2::SMAD3 | 70-83 | 13.71 | 0.95 | + | MA1622.1 | GTGGTGACTCACTG |
| RUNX2 | 160-168 | 11.84 | 0.96 | + | MA0511.2 | AAACCACAG |
| RUNX2 | 485-493 | 7.37 | 0.91 | - | MA0511.2 | ACACCACAT |
| SMAD4 | 838-845 | 13.44 | 1.00 | - | MA1153.1 | TGTCTAGA |
| SMAD1 | 1173-1186 | 11.24 | 0.90 | + | UN0263.1 | ATTTTTATCTGTTT |
| RUNX2 | 1733-1741 | 11.84 | 0.96 | - | MA0511.2 | AAACCACAG |
| **Quail** | | | | | | |
| SMAD2::SMAD3 | 72-85 | 11.96 | 0.92 | - | MA1622.1 | ACGATGAGTCACCA |
| SMAD2::SMAD3 | 73-86 | 14.80 | 0.97 | + | MA1622.1 | CTGGTGACTCATCG |
| RUNX2 | 163-171 | 11.84 | 0.96 | + | MA0511.2 | AAACCACAG |
| RUNX2 | 869-877 | 10.94 | 0.95 | + | MA0511.2 | AAACCACAT |
| SMAD3_1 | 1038-1054 | 11.71 | 0.91 | + | PB0060.1 | TGAATTCAGACAAAACA |
| RUNX2 | 1089-1097 | 9.74 | 0.94 | - | MA0511.2 | AAACCTCAA |
| SMAD4 | 1783-1790 | 10.03 | 0.96 | - | MA1153.1 | GGTCTGGA |
