# Supplementary material for: Species-specific sensitivity to TGFβ signaling and changes to the Mmp13 promoter underlie avian jaw development and evolution
Source: eLife. 2022 Jun 6;11:e66005. doi: 10.7554/eLife.66005 (PMC9246370; doi:10.7554/eLife.66005)
Supplement: Transparent reporting form [file elife-66005-transrepform1.docx]

***eLife’s* transparent reporting form**

We encourage authors to provide detailed information *within their submission* to facilitate the interpretation and replication of experiments. Authors can upload supporting documentation to indicate the use of appropriate reporting guidelines for health-related research (see [EQUATOR Network](http://www.equator-network.org/%20)), life science research (see the [BioSharing Information Resource](https://biosharing.org/)), or the [ARRIVE guidelines](http://www.plosbiology.org/article/info:doi/10.1371/journal.pbio.1000412) for reporting work involving animal research. Where applicable, authors should refer to any relevant reporting standards documents in this form.

If you have any questions, please consult our Journal Policies and/or contact us: [editorial@elifesciences.org](mailto:editorial@elifesciences.org).

**Sample-size estimation**

- You should state whether an appropriate sample size was computed when the study was being designed
- You should state the statistical method of sample size computation and any required assumptions
- If no explicit power analysis was used, you should describe how you decided what sample (replicate) size (number) to use

Please outline where this information can be found within the submission (e.g., sections or figure legends), or explain why this information doesn’t apply to your submission:

Information on sample sizes can be found in the Figure legends. Sample sizes were based on the numbers used in previously published work and include a minimum of 4-12 embryos depending on the experiment. For in vitro experiments, we used an n of 8-24 and each experiment was performed independently at least 3 times. Power calculations were not used to determine sample size.

**Replicates**

- You should report how often each experiment was performed
- You should include a definition of biological versus technical replication
- The data obtained should be provided and sufficient information should be provided to indicate the number of independent biological and/or technical replicates
- If you encountered any outliers, you should describe how these were handled
- Criteria for exclusion/inclusion of data should be clearly stated
- High-throughput sequence data should be uploaded before submission, with a private link for reviewers provided (these are available from both GEO and ArrayExpress)

Please outline where this information can be found within the submission (e.g., sections or figure legends), or explain why this information doesn’t apply to your submission:

Information on how each experiment was performed and replicated is found in the Materials and Methods section. For *in ovo* data, n refers to the total number of embryos analyzed per group. For *in vitro* data, n refers to the total number of individual wells analyzed per group, with each experiment replicated at least three times. For qPCR and luciferase experiments, each sample was run in technical duplicate and averaged. Group size “n” is denoted in the figure legends. If we encountered outliers within data sets, a standard Q-test was performed with no more than 1 outlier removed from any group. This information is found in the Material and Methods section. Criteria for exclusion of samples, such as in the TRAP quantification, are described in the Materials and Methods section.

**Statistical reporting**

- Statistical analysis methods should be described and justified
- Raw data should be presented in figures whenever informative to do so (typically when N per group is less than 10)
- For each experiment, you should identify the statistical tests used, exact values of N, definitions of center, methods of multiple test correction, and dispersion and precision measures (e.g., mean, median, SD, SEM, confidence intervals; and, for the major substantive results, a measure of effect size (e.g., Pearson's r, Cohen's d)
- Report exact p-values wherever possible alongside the summary statistics and 95% confidence intervals. These should be reported for all key questions and not only when the p-value is less than 0.05.

Please outline where this information can be found within the submission (e.g., sections or figure legends), or explain why this information doesn’t apply to your submission:

Information on statistical reporting can be found in the Statistics section of the Materials and Methods. Statistical significance was determined through two-tailed ANOVA adjusted for multiple comparisons using the Bonferroni method for the majority of experimental data, or a paired Student’s t-test for comparisons between control and treatment groups for TRAP quantification. Mean and SEM are shown for each figure. In all figures, p < 0.05 was considered statistically significant, although some statistical comparisons reached significance below p < 0.01, p < 0.001, or p < 0.0001 as noted. Exact p-values are reported in the Results section.

(For large datasets, or papers with a very large number of statistical tests, you may upload a single table file with tests, Ns, etc., with reference to sections in the manuscript.)

**Group allocation**

- Indicate how samples were allocated into experimental groups (in the case of clinical studies, please specify allocation to treatment method); if randomization was used, please also state if restricted randomization was applied
- Indicate if masking was used during group allocation, data collection and/or data analysis

Please outline where this information can be found within the submission (e.g., sections or figure legends), or explain why this information doesn’t apply to your submission:

Information on group allocation can be found in the Materials and Methods. Cell and organ culture experiments were performed using samples that were randomized prior to dividing them into control and treatment groups. For some inhibitor experiments, control and treatment-beads were applied to the same sample (left versus right side). No masking was used during group allocation, data collection, or data analysis.

**Additional data files (“source data”)**

- We encourage you to upload relevant additional data files, such as numerical data that are represented as a graph in a figure, or as a summary table
- Where provided, these should be in the most useful format, and they can be uploaded as “Source data” files linked to a main figure or table
- Include model definition files including the full list of parameters used
- Include code used for data analysis (e.g., R, MatLab)
- Avoid stating that data files are “available upon request”

Please indicate the figures or tables for which source data files have been provided:

Uncropped Western Blots are provided for Figures 2, 3, and 4.

Specimens (n = 5) from each of the three treatment groups are provided for Figure 5.

Specimens (n = 5) from each of the three treatment groups are provided for Figure 6.
